# Comparative effectiveness of physical interventions for preventing perineal trauma during vaginal delivery: a systematic review and Bayesian network meta-analysis

**DOI:** 10.3389/fmed.2026.1794056

**Published:** 2026-04-07

**Authors:** Linli Xu, Tiantian Li, Jing Jin, Li Zhang, Zhuodi Luo

**Affiliations:** 1 Department of Obstetrics, Guangdong Women and Children Hospital, Guangzhou, China; 2 Department of Dermatology, Guangdong Provincial Maternal and Child Health Care Hospital, Guangzhou, Guangdong, China

**Keywords:** episiotomy, network meta-analysis, pelvic floor exercise, perineal massage, perineal trauma, physical interventions, vaginal delivery, warm compresses

## Abstract

**Objective:**

Perineal trauma during vaginal delivery affects most primiparous women, causing significant maternal morbidity including chronic pain, sexual dysfunction, and impaired quality of life. While various physical interventions have been proposed for prevention, their comparative effectiveness remains unclear due to limited head-to-head trials. This Bayesian network meta-analysis systematically compared the efficacy and safety of all available physical interventions for preventing perineal trauma during vaginal delivery.

**Methods:**

We searched PubMed, Web of Science, Embase, and Cochrane CENTRAL for randomized controlled trials comparing physical interventions including massage, warm compresses, exercise, hands-on/off techniques, lubrication, education, or their combinations for perineal protection. Two independent reviewers screened studies, extracted data, and assessed methodological quality using the Cochrane Risk of Bias tool version 2.0. Bayesian network meta-analyses were performed using R Studio. Surface under the cumulative ranking curve (SUCRA) values were calculated to rank interventions.

**Results:**

Thirty-one randomized controlled trials involving 10,745 participants across 15 countries were included, of whom the majority were primiparous women with term singleton pregnancies enrolled in high-resource settings. For overall perineal laceration prevention, antenatal pelvic floor exercise ranked first (RR = 0.50, 95%CrI 0.28–0.87; SUCRA = 86.58%); for episiotomy prevention, massage combined with warm compresses ranked first (RR = 0.53, 95%CrI 0.28–0.93; SUCRA = 90.08%); for intact perineum promotion, massage demonstrated statistically significant superiority (RR = 1.93, 95%CrI 1.16–3.29); for severe perineal pain reduction, warm compresses had the highest SUCRA probability (SUCRA = 74.51%), though pain findings are exploratory given sparse network structures and wide credible intervals. All physical interventions demonstrated excellent neonatal safety. Education-only interventions showed no protective effects (RR = 1.03).

**Conclusion:**

Antenatal pelvic floor exercise represents the optimal first-line prevention strategy with 50% risk reduction. For women without prior training, warm compresses and intrapartum massage provide effective alternatives. Findings for pain outcomes should be interpreted as exploratory. Broader implementation of evidence-based physical interventions as evidence-informed strategies could help reduce the burden of perineal trauma, particularly among primiparous women in well-resourced settings, though generalizability to multiparous women and low-resource environments requires further investigation.

**Systematic review registration:**

https://www.crd.york.ac.uk/PROSPERO/, identifier CRD42025633266.

## 1 Introduction

Perineal trauma during vaginal delivery represents a significant public health concern, affecting approximately 85% of primiparous women worldwide (1, 2). A recent prospective investigation found that globally, the incidence of pelvic floor trauma in primiparous patients exceeds 91%, with second-degree perineal laceration occurring in approximately 40% of primiparous women, while third- and fourth-degree laceration happen in around 6% of births within this group (3). The spectrum of injury ranges from first-degree lacerations (perineal skin only) to severe third- or fourth-degree obstetric anal sphincter injuries (OASIS) involving the anal sphincter complex and rectal mucosa (4). These injuries are associated with considerable maternal morbidity, including chronic perineal pain, dyspareunia, urinary and fecal incontinence, substantially impairing postpartum quality of life and psychological well-being (5, 6). Beyond immediate physical consequences, perineal trauma imposes substantial economic burdens through emergency repairs, prolonged hospitalizations, and long-term sequelae management (7).

While routine episiotomy rates have declined in developed countries following evidence of no protective benefit against severe trauma (8, 9), controversy persists regarding optimal perineal management during the second stage of labor. Physical interventions—including perineal massage, warm compresses, hands-on/off techniques, lubrication, antenatal pelvic floor exercises, and prenatal education programs—have been proposed as beneficial strategies to reduce perineal trauma and optimize maternal outcomes (10, 11).

Several randomized controlled trials and conventional pairwise meta-analyses have investigated individual physical interventions with conflicting results. Some studies reported that intrapartum perineal massage significantly reduced the risk of perineal trauma requiring suturing and episiotomy rates (12, 13), while others found no significant protective effects against specific laceration degrees. Similarly, warm compresses have demonstrated variable efficacy across studies, with systematic reviews showing substantial reductions in third- and fourth-degree lacerations when combined with perineal massage compared to control groups (14). Hands-on techniques (manual perineal support and controlled fetal head delivery) versus hands-off approaches (minimal intervention allowing spontaneous delivery) have yielded inconsistent findings regarding perineal protection, with recent meta-analyses indicating that hands-off techniques may reduce episiotomy rates without increasing severe perineal trauma (15). The heterogeneity in study designs, intervention protocols, outcome definitions, and participant populations has precluded definitive conclusions regarding the comparative effectiveness of these diverse strategies.

Traditional pairwise meta-analyses can only synthesize evidence from head-to-head comparisons between two interventions, limiting their ability to compare multiple competing interventions simultaneously and rank their relative effectiveness (16). Network meta-analysis (NMA) overcomes this limitation by integrating both direct evidence (from head-to-head trials) and indirect evidence (derived through common comparators) within a unified analytical framework, enabling comprehensive comparison of all available interventions and probabilistic ranking of their efficacy (17, 18). This methodology is particularly valuable for clinical decision-making when multiple treatment options exist without complete head-to-head comparison data.

To date, network meta-analysis remains limited in the field of perineal trauma prevention, with insufficient comprehensive evidence comparing all available physical interventions for perineal protection during vaginal delivery. Previous systematic reviews have examined specific intervention categories in isolation or have been limited to pairwise comparisons (19–21), failing to provide an integrated evidence synthesis that would inform optimal clinical practice. A recent network meta-analysis of episiotomy approaches highlighted the importance of comparing multiple strategies simultaneously for comprehensive understanding of maternal and neonatal outcomes (22). Given the clinical importance of perineal trauma prevention and the expanding array of proposed physical interventions, a rigorous network meta-analysis is urgently needed to establish the comparative effectiveness and safety profile of these approaches. Therefore, we conducted this comprehensive Bayesian network meta-analysis to systematically compare the efficacy and safety of all available physical interventions—including massage, warm compresses, exercise, hands-on/off techniques, lubrication, education, and their combinations—in preventing perineal trauma during vaginal delivery, establish hierarchical rankings of intervention effectiveness, and provide evidence-based recommendations to guide clinical practice and optimize maternal perineal outcomes.

## 2 Methods

### 2.1 Study design

This network meta-analysis was prospectively registered with the International Prospective Register of Systematic Reviews (PROSPERO) (registration number: CRD42025633266). The systematic review was conducted according to the Preferred Reporting Items for Systematic Reviews and Meta-Analyses (PRISMA) extension statement for network meta-analyses (23). A completed PRISMA checklist confirming adherence to all applicable reporting items is provided in Supplementary Table S2.

### 2.2 Search strategy

Two researchers independently searched four electronic databases: PubMed, Web of Science, Embase, and the Cochrane Central Register of Controlled Trials (CENTRAL) from January 1, 2001, to November 30, 2025. The search strategy combined terms related to perineal trauma (“perineal injury” OR “perineal damage” OR “perineal trauma” OR “perineal tear” OR “perineal laceration” OR “perineal rupture”) and interventions (massage, warm compress, exercise, hands-on technique, hands-off technique, lubrication, education, physical intervention, perineal protection). Medical Subject Headings (MeSH) terms and free-text words were used in combination to maximize search sensitivity. The search was limited to randomized controlled trials without language restrictions. Search strategies are provided in full in Supplementary Table S1. Additionally, reference lists of included studies and relevant systematic reviews were manually searched to identify additional eligible studies.

### 2.3 Inclusion and exclusion criteria

The inclusion of studies meeting the criteria was based on the PICOS framework:

*Population*: pregnant women undergoing vaginal delivery, with preference for primiparous women with singleton pregnancies at ≥37 weeks gestation, without restriction to maternal age or ethnicity.
*Intervention*: physical interventions for perineal protection including perineal massage (antenatal or intrapartum), warm compresses, exercise (antenatal pelvic floor muscle training), hands-on techniques (manual perineal support and controlled fetal head delivery), hands-off techniques (minimal intervention allowing spontaneous delivery), lubrication (oils or gels), prenatal education programs, or combinations thereof. There were no restrictions on the frequency, duration, or specific protocols of the above interventions.
*Comparison*: routine care, placebo, or other active physical interventions.
*Outcomes*: primary outcome was overall perineal laceration rate. Secondary outcomes included laceration severity grades (first-degree, second-degree, third- or fourth-degree), episiotomy rate, intact perineum rate, perineal pain (mild, moderate, or severe), and neonatal outcomes (Apgar scores at 1 and 5 min).
*Study design*: randomized controlled trials only. Non-randomized studies (cohort studies, case–control studies, case series, case reports), conference abstracts, letters, editorials, reviews, systematic reviews, meta-analyses, studies without a control group, studies with pharmacological interventions as primary comparisons, duplicate publications or overlapping cohorts (the most recent or comprehensive publication was retained), studies with insufficient data for extraction or analysis, and studies with sample sizes fewer than 10 participants per arm were excluded.

Based on the criteria set above, two authors independently screened the titles and abstracts to exclude duplicates and studies that did not meet the inclusion criteria. Subsequently, the eligible studies were reviewed in full text. Any inconsistencies that arose during this period were resolved through discussion or consultation with a third reviewer.

### 2.4 Literature screening and data extraction

All retrieved records were imported into EndNote reference management software, and duplicates were removed. Two independent reviewers screened titles and abstracts against the eligibility criteria, followed by full-text assessment of potentially eligible studies. Following the Cochrane Handbook for Systematic Reviews of Interventions, data extraction was performed independently by two reviewers using a standardized, pre-piloted data extraction form (24). The extracted information included basic publication information (first author, publication year, country, trial registration number), participant characteristics (total sample size, parity distribution, gestational age, maternal age), intervention details (type of physical intervention, timing, duration, frequency, technique description, comparator, and co-interventions), and outcome data. For dichotomous outcomes including perineal laceration, episiotomy, intact perineum, and pain levels, the number of events and total participants in each arm were extracted. For continuous outcomes such as Apgar scores, means, standard deviations, and sample sizes were extracted. For studies with multiple intervention arms, all relevant pairwise comparisons were extracted. When necessary data were not reported in the published article, study authors were contacted for additional information. Any disagreements were resolved through discussion or consultation with a third reviewer.

The methodological quality of included RCTs was assessed by two independent reviewers using the Cochrane Risk of Bias tool version 2.0 (RoB 2) (25), which evaluated bias arising from the randomization process, deviations from intended interventions, missing outcome data, outcome measurement, and selection of reported results. Each domain was rated as low risk, some concerns, or high risk according to the signaling questions and algorithms specified in the RoB 2 guidance. The certainty of evidence for each outcome was assessed using the Grading of Recommendations Assessment, Development and Evaluation (GRADE) approach for network meta-analysis. Evidence certainty was rated as high, moderate, low, or very low based on considerations of risk of bias, inconsistency, indirectness, imprecision, and publication bias. The assessment was performed in Review Manager 5.4 and GRADEpro GDT software.

### 2.5 Statistical analysis

Bayesian network meta-analyses were performed using R Studio with the gemtc and BUGSnet packages (26). Both fixed-effects and random-effects models were fitted, with model selection based on deviance information criterion (DIC)—lower values indicating superior fit, with differences ≥3–5 points considered meaningful. For dichotomous outcomes, relative risks (RR) with 95% credible intervals (CrI) were calculated; for continuous outcomes, mean differences (MD) with 95% CrIs were computed. Routine care served as the reference comparator. Results were deemed significant when 95% CrIs excluded the null value (RR = 1.0 or MD = 0). Network diagrams visualized evidence structure, with node sizes reflecting participant numbers and edge thickness indicating study counts. Inconsistency between direct and indirect evidence was evaluated by comparing consistency versus inconsistency models; DIC differences <3–5 points indicated acceptable consistency. For closed loops, node-splitting analysis assessed loop-specific inconsistency (*p* > 0.05 indicating consistency). Treatment rankings were determined by calculating surface under the cumulative ranking curve (SUCRA) values (0–100%, higher indicating better performance) (27). SUCRA curves visualized probability rankings across interventions. MCMC simulations employed three chains with 50,000 iterations after 20,000 burn-in iterations, thinned by 10. Convergence was verified through trace plots and Gelman-Rubin diagnostics. Zero-event arms received 0.5 continuity corrections. For outcomes with statistically significant summary estimates, 95% predictive intervals were calculated from the posterior distribution of the between-study standard deviation (*τ*) to characterise the expected range of true effects in a new study, accounting for between-study heterogeneity (τ^2^). All analyses were two-sided with *α* = 0.05, reported per PRISMA-NMA standards (28).

## 3 Results

### 3.1 Literature search and study characteristics

**Figure 1:**
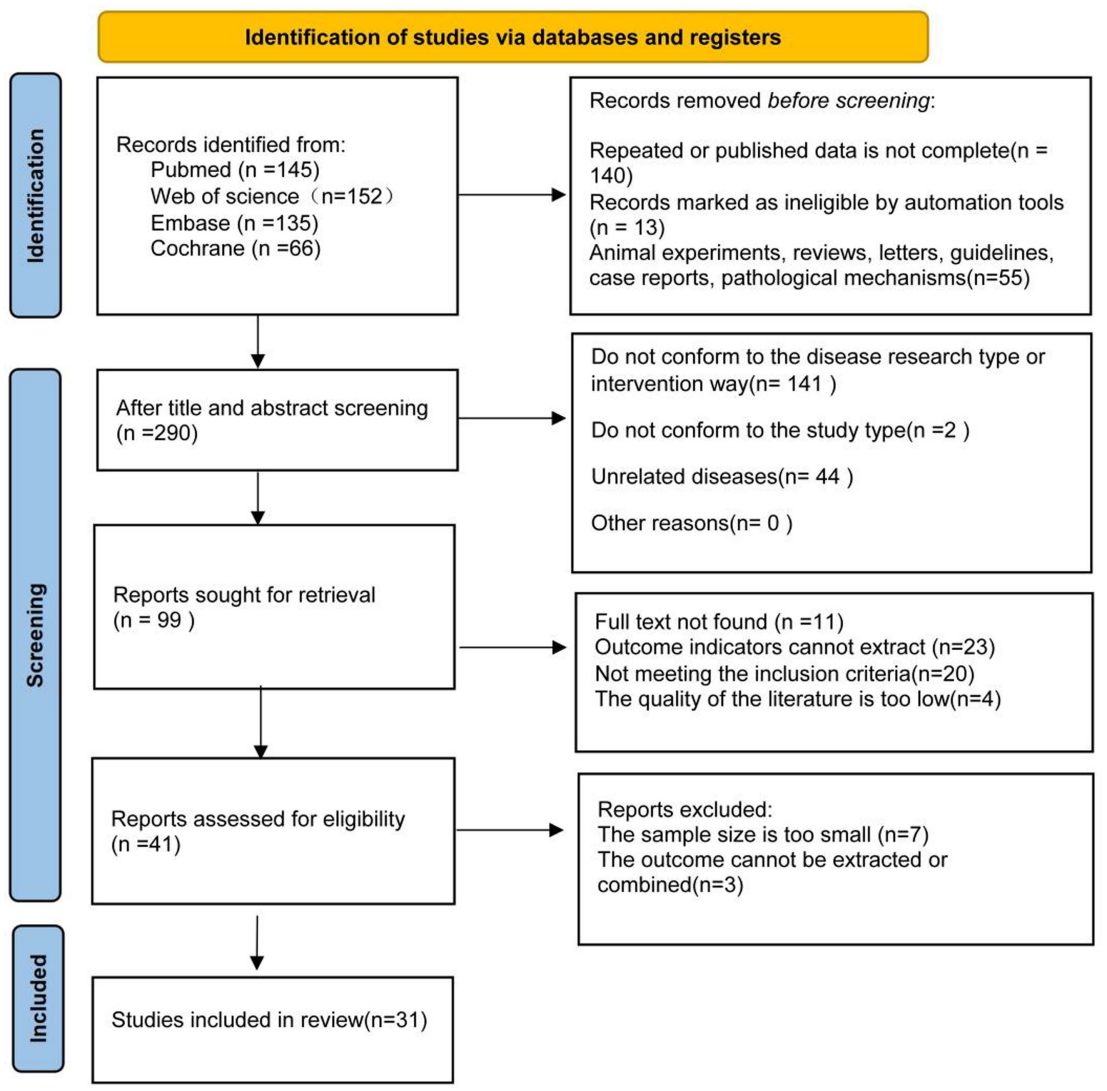
Literature screening process.

The systematic search identified 498 records from four databases. After removing 208 duplicates and ineligible records, 290 records underwent screening. Subsequently, 187 records were excluded during title and abstract screening, and 68 reports were excluded after full-text assessment due to unavailability, inability to extract outcomes, or failure to meet inclusion criteria. Ultimately, 31 randomized controlled trials were included in this network meta-analysis (Figure 1).

The 31 included randomized controlled trials, published between 2001 and 2025, enrolled 10,745 participants across 15 countries. Most studies (*n* = 25) recruited exclusively primiparous women with singleton pregnancies at ≥37 weeks gestation, and the majority were conducted in high-resource settings (*n* = 23, 74.2%), including Europe, North America, and East Asia. Physical interventions evaluated comprised 11 distinct approaches including massage, warm compresses, exercise, hands-on/off techniques, lubrication, and their combinations. Detailed baseline characteristics are summarized in Table 1.

**Table 1:** Characteristics of included studies.

Author	Year	Country	Study type	Intervention mode		Number of cases		Age years		Gestational week		Birth weight (g)		Inclusion criteria	Outcome indicators
				Experimental group	Control group	Experimental group	Control group	Experimental group	Control group	Experimental group	Control group	Experimental group	Control group		
Stamp (65)	2001	Australia	RCT	Lubricated perineal massage	Routine care	708	632	NA	NA	> 36	> 36	NA	NA	Primiparous women with ≥37 weeks gestation, singleton pregnancy, cephalic presentation, healthy pregnancies.	1, 2, 3, 4, 5
Albers (66)	2005	USA	RCT	Warm Compresses	Routine care	404	404	24.90 ± 5.30	24.50 ± 5.10	> 37	> 37	3351.00 ± 437.00	3345.00 ± 440.00	Primiparous women with ≥37 weeks gestation, singleton pregnancy, cephalic presentation, healthy pregnancies.	1, 2, 3, 4, 5
Albers (66)	2005	USA	RCT	Lubricated perineal massage	Routine care	403	404	24.50 ± 5.20	24.50 ± 5.10	> 37	> 37	3349.00 ± 462.00	3345.00 ± 440.00	Primiparous women with ≥37 weeks gestation, singleton pregnancy, cephalic presentation, healthy pregnancies.	1, 2, 3, 4, 5
Costa (67)	2006	Brazil	RCT	Hands on	Routine care	35	35	20.10 ± 3.30	18.60 ± 2.70	38.58 ± 0.99	38.59 ± 0.87	3017.70 ± 416.00	2996.70 ± 334.60	≥37 weeks gestation with singleton pregnancy, cephalic presentation.	1, 2, 3, 4, 5
Dahlen (68)	2007	Australia	RCT	Warm compression	Routine care	360	357	27.00 ± 5.50	27.20 ± 4.90	> 36	> 36	3365.00 ± 447.00	3346.00 ± 450.00	Primiparous or multiparous women with ≥37 weeks gestation, singleton pregnancy, cephalic presentation, healthy pregnancies.	1, 2, 3, 4, 5
Mei-dan (69)	2008	Israel	RCT	Perineal massage and warm compression	Routine care	128	106	27.60 ± 3.50	25.40 ± 3.80	39.30 ± 1.30	38.90 ± 1.50	3237.00 ± 394.00	3130.00 ± 434.00	Primiparous women with term pregnancy, singleton pregnancy, cephalic presentation, healthy pregnancies.	1, 2, 3, 4, 5
Araújo (70)	2008	Brazil	RCT	Lubricated by Liquid Vaseline	Routine care	38	38	21.60 ± 3.80	20.50 ± 3.90	39.30 ± 1.30	38.90 ± 1.50	3237.37 ± 384.70	3.00 ± 320.20	Primiparous women with ≥37 weeks gestation, singleton pregnancy, cephalic presentation, healthy pregnancies.	1, 2, 3, 4, 5
Schaub (71)	2008	Switzerland	RCT	Obstetric gel	Routine care	94	89	28.60 ± 5.27	28.60 ± 3.81	40.05 ± 0.93	40.08 ± 1.07	3433.70 ± 454.90	3384.90 ± 388.15	Primiparous women with ≥37 weeks gestation, singleton pregnancy, cephalic presentation, healthy pregnancies.	2, 3, 4, 5
Ibrahim (72)	2017	Egypt	RCT	Warm compression	Routine care	102	100	23.76 ± 6.21	24.78 ± 5.57	38.25 ± 3.95	39.00 ± 1.83	3050.00 ± 212.00	3010.00 ± 282.00	Primiparous women with ≥37 weeks gestation, singleton pregnancy, cephalic presentation, healthy pregnancies.	2, 3, 4, 5
Foroughipour (73)	2011	Iran	RCT	Hands-on	Routine care and education	50	50	24.70 ± 3.83	25.20 ± 5.04	NA	NA	NA	NA	Primiparous women with ≥37 weeks gestation, singleton pregnancy, cephalic presentation, healthy pregnancies.	1, 2, 3, 4, 5
Rezaei (74)	2014	Iran	RCT	Hands on	Routine care	300	300	22.40 ± 2.90	22.70 ± 3.01	NA	NA	3125.09 ± 328.10	3163.02 ± 387.40	Primiparous women with ≥37 weeks gestation, singleton pregnancy, cephalic presentation, planning vaginal delivery, healthy pregnancies.	1, 2, 3, 4, 5
Zare (75)	2014	Iran	RCT	Lubricated perineal massage	Routine care	100	45	26.96 ± 4.30	26.06 ± 4.50	38.84 ± 1.03	38.67 ± 0.94	3348.00 ± 452.00	3280.00 ± 407.00	Primiparous women with ≥37 weeks gestation, singleton pregnancy, cephalic presentation, healthy pregnancies.	2, 3, 4, 5
Demirel and Golbasi (60)	2015	Turkey	RCT	Perineal massage	Routine care	142	142	24.30 ± 4.09	23.42 ± 3.74	NA	NA	NA	NA	Primiparous or multiparous women with ≥37 weeks gestation, singleton pregnancy, cephalic presentation.	1, 2, 3, 4, 5
Dönmez (76)	2015	Turkey	RCT	Perineal massage	Routine care	30	39	26.90 ± 4.64	24.25 ± 4.15	39.16 ± 1.05	38.66 ± 2.14	3038.00 ± 300.24	3175.25 ± 583.37	Primiparous women with ≥37 weeks gestation, singleton pregnancy, cephalic presentation, healthy pregnancies.	1, 2, 3, 4, 5
Dönmez (76)	2015	Turkey	RCT	Massage and Kegel Exercises	Routine care	32	39	28.03 ± 4.15	24.25 ± 4.15	39.18 ± 1.33	38.66 ± 2.14	3213.43 ± 430.84	3175.25 ± 583.37	Primiparous women with ≥37 weeks gestation, singleton pregnancy, cephalic presentation, healthy pregnancies.	1, 2, 3, 4, 5
Shahoei (77)	2015	Iran	RCT	Perineal massage and warm compression	Routine care	75	75	26.25 ± 4.35	26.42 ± 3.65	39.92 ± 0.84	39.34 ± 0.80	3000.00 ± 760.00	3000.00 ± 570.00	Primiparous women with ≥37 weeks gestation, singleton pregnancy, cephalic presentation, healthy pregnancies.	1, 2, 3, 4, 5
Essa (78)	2015	Egypt	RCT	Warm compresses	Routine care	80	80	NA	NA	NA	NA	NA	NA	Primiparous women with ≥37 weeks gestation, singleton pregnancy, cephalic presentation, healthy pregnancies.	2, 3, 4, 5
Shahoei (79)	2017	Iran	RCT	Lubricated perineal massage	Routine care	90	83	25.62 ± 4.25	25.31 ± 3.86	39.00 ± 0.93	39.00 ± 0.97	3100.00 ± 852.00	3100.00 ± 852.00	Primiparous women with ≥37 weeks gestation, singleton pregnancy, cephalic presentation, planning vaginal delivery, healthy pregnancies.	1, 2, 3, 4, 5
Leon-Larios et al. (64)	2017	Spain	RCT	Perineal massage and pelvic floor exercises	Routine care	193	160	32.18 ± 4.02	29.56 ± 5.17	NA	NA	281.93 (410.86)	3237.67 (439.4)	Primiparous women with term pregnancy, singleton pregnancy, cephalic presentation, healthy pregnancies.	3, 5
Akhlaghi (80)	2019	Iran	RCT	Perineal massage and warm compression	Routine care	50	49	22.46 ± 3.94	23.87 ± 4.86	40.0 (37.0, 42.0)	40.00 (38.0, 42.0)	3250.00 ± 362.93	3160.00 ± 428.15	Primiparous women with ≥37 weeks gestation, singleton pregnancy, cephalic presentation, healthy pregnancies.	1, 2, 3, 4, 5
Dieb et al. (56)	2020	Egypt	RCT	Perineal massage and PFMT education	Routine care and education	200	200	38.29 ± 1.90	37.90 ± 3.47	38.54 ± 1.62	38.66 ± 2.08	3030.00 ± 168.85	3050.00 ± 191.10	Primiparous or multiparous women with ≥37 weeks gestation, singleton pregnancy, cephalic presentation, healthy pregnancies.	1, 2, 3, 4, 5
Romina (81)	2020	Iran	RCT	Perineal massage and warm compression	Routine care	39	38	24.18 ± 4.20	25.38 ± 3.65	39.35 ± 1.06	39.42 ± 0.97	NA	NA	Primiparous women with ≥37 weeks gestation, singleton pregnancy, cephalic presentation, healthy pregnancies.	1, 2, 3, 4, 5
Modoor et al. (49)	2021	Saudi Arabia	RCT	Warm compression	Routine care	50	50	24.60 ± 3.90	24.52 ± 3.60	38.94 ± 1.15	39.28 ± 1.11	NA	NA	Primiparous women with ≥37 weeks gestation, singleton pregnancy, cephalic presentation, planning vaginal delivery, healthy pregnancies.	1, 2, 3, 4, 5
Silva-Jose et al. (59)	2021	Spain	RCT	Exercise	Routine care	48	50	33.15 ± 4.82	33.54 ± 4.87	NA	NA	NA	NA	Term pregnancy with singleton pregnancy, healthy pregnancies.	2, 3, 5
Faraz (82)	2022	UAE	RCT	Hands on and warm compression	Hands on	99	93	NA	NA	38.97 ± 1.26	39.24 ± 1.25	NA	NA	Primiparous women with ≥37 weeks gestation, singleton pregnancy, cephalic presentation, healthy pregnancies.	2, 3, 4, 5
Azarkish (83)	2022	Iran	RCT	Lubricant gel	Routine care	82	81	22.53 ± 6.68	23.07 ± 6.89	39.53 ± 0.83	39.27 ± 1.00	2851.82 ± 344.26	2968.73 ± 503.09	Primiparous women with ≥37 weeks gestation, singleton pregnancy, cephalic presentation, healthy pregnancies.	2, 3, 4, 5
He et al. (55)	2023	China	RCT	Perineal massage	Routine care and education	43	48	31.10 ± 2.40	30.80 ± 3.20	39.70 (38.90, 40.6)	39.2 (38.40, 40.10)	3290.80 ± 377.40	3303.40 ± 390.30	Primiparous women with term pregnancy, singleton pregnancy, planning vaginal delivery, healthy pregnancies.	1, 2, 4, 5
He et al. (55)	2023	China	RCT	PFMT	Routine care and education	48	48	31.40 ± 3.10	30.80 ± 3.20	39.60 (39.00, 40.10)	39.20 (38.40, 40.10)	3324.90 ± 466.00	3303.40 ± 390.30	Primiparous women with term pregnancy, singleton pregnancy, planning vaginal delivery, healthy pregnancies.	1, 2, 4, 5
He et al. (55)	2023	China	RCT	Perineal massage and PFMT	Routine care and education	48	48	31.20 ± 2.70	30.80 ± 3.20	39.80 (38.70, 40.40)	39.20 (38.40, 40.10)	3243.10 ± 390.30	3303.40 ± 390.30	Primiparous women with term pregnancy, singleton pregnancy, planning vaginal delivery, healthy pregnancies.	1, 2, 4, 5
Rodrigues et al. (45)	2023	Portugal	RCT	Perineal massage and warm compression	Hands on	400	400	31.80 ± 5.10	31.40 ± 4.80	39.30 ± 1.10	39.30 ± 1.10	3345.00 ± 385.00	3328.00 ± 400.00	Primiparous women with ≥37 weeks gestation, singleton pregnancy, cephalic presentation, planning vaginal delivery, healthy pregnancies.	1, 2, 3, 4, 5
Bqlein (84)	2024	Saudi Arabia	RCT	Warm compression	Routine care	40	40	30.75 ± 4.18	31.50 ± 3.72	≥ 37	≥ 37	NA	NA	Primiparous or multiparous women with ≥37 weeks gestation, singleton pregnancy.	2, 3, 4, 5
Labrecque (85)	2000	Canada	RCT	Perineal massage	Routine care	519	515	28.00 ± 4.90	27.90 ± 4.70	33.10 ± 1.20	33.00 ± 1.30	NA	NA	Primiparous women with term pregnancy, singleton pregnancy, cephalic presentation, planning vaginal delivery, healthy pregnancies.	1, 2, 3, 4, 5
Labrecque (85)	2000	Canada	RCT	Perineal massage	Routine care	246	247	31.10 ± 4.10	30.70 ± 4.50	33.00 ± 1.40	32.90 ± 1.30	NA	NA	Multiparous women with term pregnancy, singleton pregnancy, cephalic presentation, planning vaginal delivery, healthy pregnancies.	1, 2, 3, 4, 5
Taylor and Stulz (54)	2024	Australia	RCT	Perineal myofascial release manipulation	routine care	50	49	28.10 ± 5.30	28.50 ± 4.70	39.10 ± 1.20	39.30 ± 1.40	NA	NA	Primiparous women with ≥37 weeks gestation, singleton pregnancy, cephalic presentation, healthy pregnancies.	1, 2, 3, 4, 5
Guo et al. (57)	2025	China	RCT	Perineal massage and warm compression	routine care	82	82	28.30 ± 3.10	28.20 ± 2.90	39.60 ± 0.90	39.60 ± 0.90	3207.10 ± 326.10	3257.50 ± 324.20	Primiparous women with ≥37 weeks gestation, singleton pregnancy, cephalic presentation, healthy pregnancies.	1, 3, 5
Nnabuchi et al. (44)	2025	Nigeria	RCT	Perineal massage and warm compression	routine care	94	89	NA	NA	38.00 ± 1.30	37.80 ± 1.20	3400.00 ± 200.00	3400.00 ± 300.00	≥37 weeks gestation with singleton pregnancy, healthy pregnancies.	1, 3, 4, 5

1: Intact perineum rate 2: Tear degree classification 3: Episiotomy rate 4: Pain scores 5: Apgar score.

### 3.2 Risk of bias assessment

**Figure 2:**
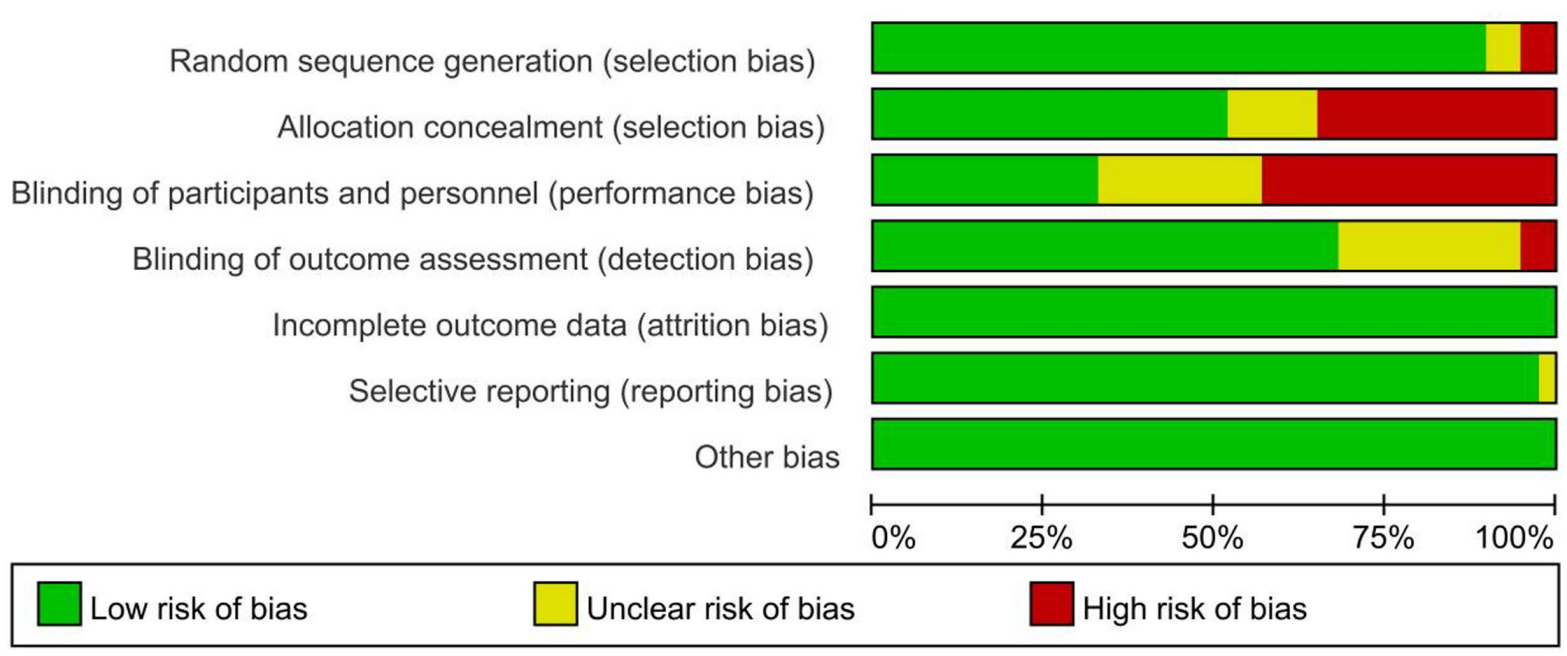
Risk of bias graph.

The methodological quality of the 31 included studies was assessed using the Cochrane Risk of Bias tool version 2.0 (Figure 2 and Supplementary Figure S1). Overall, 23 studies (74.2%) demonstrated low risk of bias across all domains, while 8 studies (25.8%) had some concerns in one or more domains. Of the 8 studies rated as ‘some concerns,’ 6 studies (Albers 2005, Albers 2005a, Costa 2006, Dahlen 2007, Schaub 2008, and Ibrahim 2017) received this rating exclusively due to Domain 2 (blinding of participants and personnel), reflecting the inherent impossibility of blinding participants and care providers in physical intervention trials. The remaining 2 studies (Foroughipour 2011 and Mei-dan 2008) had ‘some concerns’ in Domain 1 (random sequence generation) due to insufficient reporting of randomization methods, rather than evidence of inadequate randomization per se. No studies were rated as high risk of bias. Most studies showed adequate random sequence generation (90.3%) and allocation concealment, with minimal attrition (87.1%) and comprehensive outcome reporting (93.5%). The most common concerns were related to blinding of participants and personnel (19.4%), which is inherently challenging for physical interventions during childbirth. At the outcome level, the proportion of contributing studies rated as low risk of bias was as follows: overall perineal laceration, 23/31 (74.2%); first-degree laceration, 22/29 (75.9%); second-degree laceration, 22/29 (75.9%); severe laceration (third/fourth-degree), 16/20 (80.0%); episiotomy rate, 22/30 (73.3%); intact perineum, 20/26 (76.9%); perineal pain outcomes, 5–6 of 6–7 contributing studies (≥83.3%); and neonatal Apgar scores, 7/8 (87.5%), with the remainder rated as ‘some concerns’ in each case. The predominant source of concern across all outcomes was blinding of participants and personnel (Domain 2 of RoB 2), which is inherent to physical intervention research and is unlikely to introduce meaningful differential bias for the objectively assessed primary outcomes. Notably, none of the ‘some concerns’ ratings involved outcome assessment, missing data, or selective reporting, which are the domains most likely to introduce bias into effect estimates. As these 8 studies represent a minority of participants across all primary outcome networks, and as the primary outcomes comprise objectively documented clinical findings unlikely to be affected by performance or detection bias, excluding these higher-risk studies would not be expected to materially alter the direction, magnitude, or SUCRA rankings of the main results. This interpretation is further supported by the node-splitting analysis confirming consistency between direct and indirect evidence across all closed loops (all *p* > 0.05, Supplementary Figure S2).

### 3.3 GRADE evidence quality assessment

The certainty of evidence for each outcome was assessed using the GRADE approach, considering risk of bias, inconsistency, indirectness, imprecision, and publication bias (Table 2). The primary downgrading factors were inconsistency across studies and imprecision of effect estimates. No outcome was downgraded for indirectness. Regarding publication bias, comparison-adjusted funnel plots could not be reliably constructed for most comparisons due to the insufficient number of studies per direct treatment pair (fewer than 10 in most cases). Therefore, the potential for publication bias could not be formally assessed and was not used as a GRADE downgrading criterion, though it cannot be excluded as a source of bias in the current synthesis. For critical outcomes, severe perineal laceration achieved ⊕⊕⊕⊕ HIGH certainty evidence with no serious concerns. Perineal laceration was rated as ⊕⊕⊕◯ MODERATE due to serious inconsistency, while episiotomy rate was graded as ⊕⊕◯◯ LOW due to very serious inconsistency. For important outcomes, most were rated as ⊕⊕⊕◯ MODERATE due to serious inconsistency. Pain-related outcomes showed variable certainty: mild and severe perineal pain achieved ⊕⊕⊕⊕ HIGH quality evidence, while moderate pain was ⊕◯◯◯ VERY LOW due to very serious inconsistency and imprecision. Apgar scores at 1 min were supported by ⊕⊕⊕⊕ HIGH quality evidence, while 5-min scores were ⊕⊕⊕◯ MODERATE.

**Table 2:** Quality assessment.

Participants (studies)	Risk of bias	Inconsistency	Indirectness	Imprecision	Publication bias	Overall quality of evidence	Summary of findings				
							Study event rates (%)		Relative effect (95% CI)	Anticipated absolute effects	
							Control	Intervention		Risk with Control	Risk difference with Intervention (95% CI)
Perineal laceration (critical outcome)											
11,372 (35 studies)	No serious	Serious	No serious	No serious	Undetected	⊕⊕⊕◯ MODERATE due to inconsistency	2552/5727 (44.6%)	2689/5645 (47.6%)	OR 0.85 (0.78–0.93)	40 fewer per 1,000	From 61 fewer to 18 fewer
Intact perineum (important outcome)											
8,069 (25 studies)	No serious	Serious	No serious	No serious	Undetected	⊕⊕⊕◯ MODERATE due to inconsistency	1194/3985 (30.0%)	820/3885 (21.1%)	OR 1.63 (1.47–1.81)	95 more per 1,000	From 73 more to 118 more
1st degree laceration (important outcome)											
8,949 (29 studies)	No serious	Serious	No serious	No serious	Undetected	⊕⊕⊕◯ MODERATE due to inconsistency	993/4465 (22.2%)	897/4484 (20.0%)	OR 1.17 (1.05–1.30)	26 more per 1,000	From 8 more to 45 more
2nd degree laceration (important outcome)											
8,902 (29 studies)	No serious	Serious	No serious	No serious	Undetected	⊕⊕⊕◯ MODERATE due to inconsistency	750/4468 (16.8%)	885/4434 (20.0%)	OR 0.79 (0.71–0.88)	35 fewer per 1,000	From 49 fewer to 20 fewer
Severe perineal laceration (critical outcome)											
9,154 (23 studies)	No serious	No serious	No serious	No serious	Undetected	⊕⊕⊕⊕ HIGH	104/4611 (2.3%)	224/4543 (4.9%)	OR 0.44 (0.35–0.56)	27 fewer per 1,000	From 31 fewer to 21 fewer
Episiotomy rate (critical outcome)											
8,646 (30 studies)	No serious	Very serious	No serious	No serious	Undetected	⊕⊕◯◯ LOW due to inconsistency	1065/4325 (24.6%)	1349/4321 (31.2%)	OR 0.63 (0.56–0.71)	90 fewer per 1,000	From 110 fewer to 68 fewer
Perineal pain (mild) (important outcome)											
1703 (7 studies)	No serious	Serious	No serious	Serious	Undetected	⊕⊕⊕⊕ HIGH due to inconsistency, imprecision	276/859 (32.1%)	135/844 (16.0%)	OR 3.25 (2.54–4.41)	229 more per 1,000	From 160 more to 296 more
Perineal pain (moderate) (important outcome)											
1703 (7 studies)	No serious	Very serious	No serious	Serious	Undetected	⊕◯◯◯ VERY LOW due to inconsistency, imprecision	225/859 (26.2%)	188/844 (22.3%)	OR 1.24 (0.99–1.55)	39 more per 1,000	From 2 fewer to 85 more
Perineal pain (severe or more) (important outcome)											
1703 (7 studies)	No serious	Very serious	No serious	No serious	Undetected	⊕⊕⊕⊕ HIGH due to inconsistency	206/859 (24.0%)	398/844 (47.2%)	OR 0.30 (0.24–0.37)	260 fewer per 1,000	From 295 fewer to 223 fewer
1 min APGAR (important outcome)											
2,211 (9 studies)	No serious	No serious	No serious	No serious	Undetected	⊕⊕⊕⊕ HIGH	1,124	1,087	–		The mean 1 min APGAR in the intervention groups was 0 higher (−0.05 to 0.05 higher)
5 min APGAR (important outcome)											
2,260 (9 studies)	No serious	Very serious	No serious	Serious	Undetected	⊕⊕⊕◯ MODERATE due to inconsistency, imprecision	1,124	1,136	–		The mean 5 min APGAR in the intervention groups was 001 higher (0.02 to 0.04 higher)

### 3.4 Primary outcome: overall perineal laceration rate

**Figure 3:**
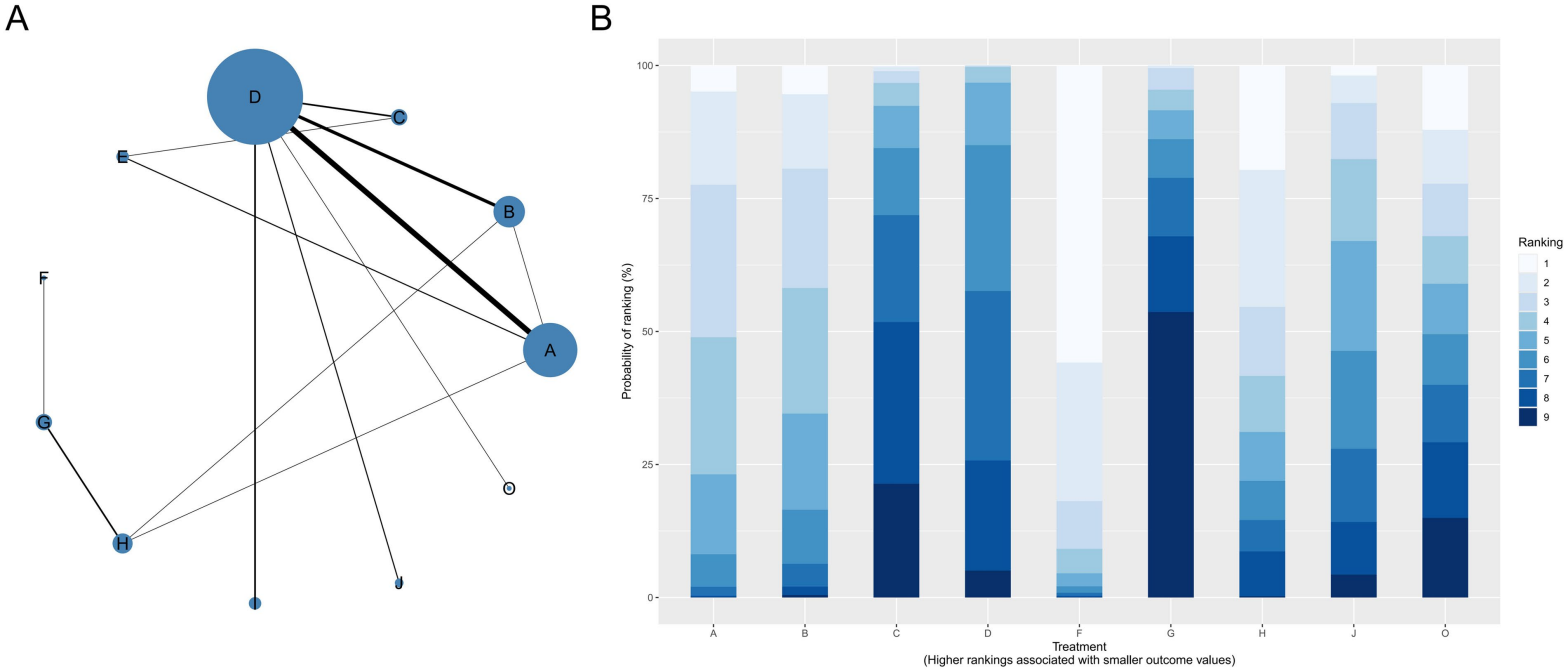
Network geometry **(A)** and SUCRA rankings **(B)** for overall perineal laceration prevention. D, Routine care; A, Massage; B, Warm compresses; C, Exercise; E, Education; F, Massage + warm compresses; G, Hands-on techniques; H, Hands-off techniques; I, Lubrication; J, Lubricated massage; O, Massage + exercise.

The network comprised 11 intervention nodes connected by 13 direct comparisons across 31 RCTs with 10,721 participants, generating 5,111 perineal laceration events (overall event rate: 47.7%). Figure 3A presents the network geometry, with node size proportional to the number of participants directly studied. The most common intervention was routine care with 25 studies (*n* = 3,198), followed by massage (14 studies, *n* = 2,767) and warm compresses (8 studies, *n* = 1,258). Model comparison based on the deviance information criterion (DIC) favored the random-effects model (DIC = 138.4) over the fixed-effects model (DIC = 188.2), indicating substantial between-study heterogeneity. The between-study standard deviation (*τ*) and τ^2^ for each outcome network are reported in Supplementary Table S3, ranging from τ = 0.153 (Apgar 5-min) to τ = 1.440 (severe perineal pain), reflecting that heterogeneity was most pronounced for sparse, subjectively assessed outcomes and least pronounced for neonatal outcomes. The consistency model versus inconsistency model showed minimal difference (1.3 points), indicating no substantial inconsistency between direct and indirect evidence (all *p*-values > 0.05, Supplementary Figure S1). Compared to routine care, exercise demonstrated statistically significant protective effects [RR = 0.50, 95%CrI (0.28, 0.87)], representing a 50% risk reduction in overall perineal laceration. Other interventions showing protective trends included warm compresses [RR = 0.72, 95%CrI (0.49, 1.02)], lubrication [RR = 0.72, 95%CrI (0.37, 1.34)], hands-on techniques [RR = 0.73, 95%CrI (0.31, 1.69)], hands-off techniques [RR = 0.78, 95%CrI (0.39, 1.51)], and massage [RR = 0.82, 95%CrI (0.62, 1.09)]. Education alone [RR = 1.03, 95%CrI (0.55, 2.00)] showed no protective effects.

**Figure 4:**
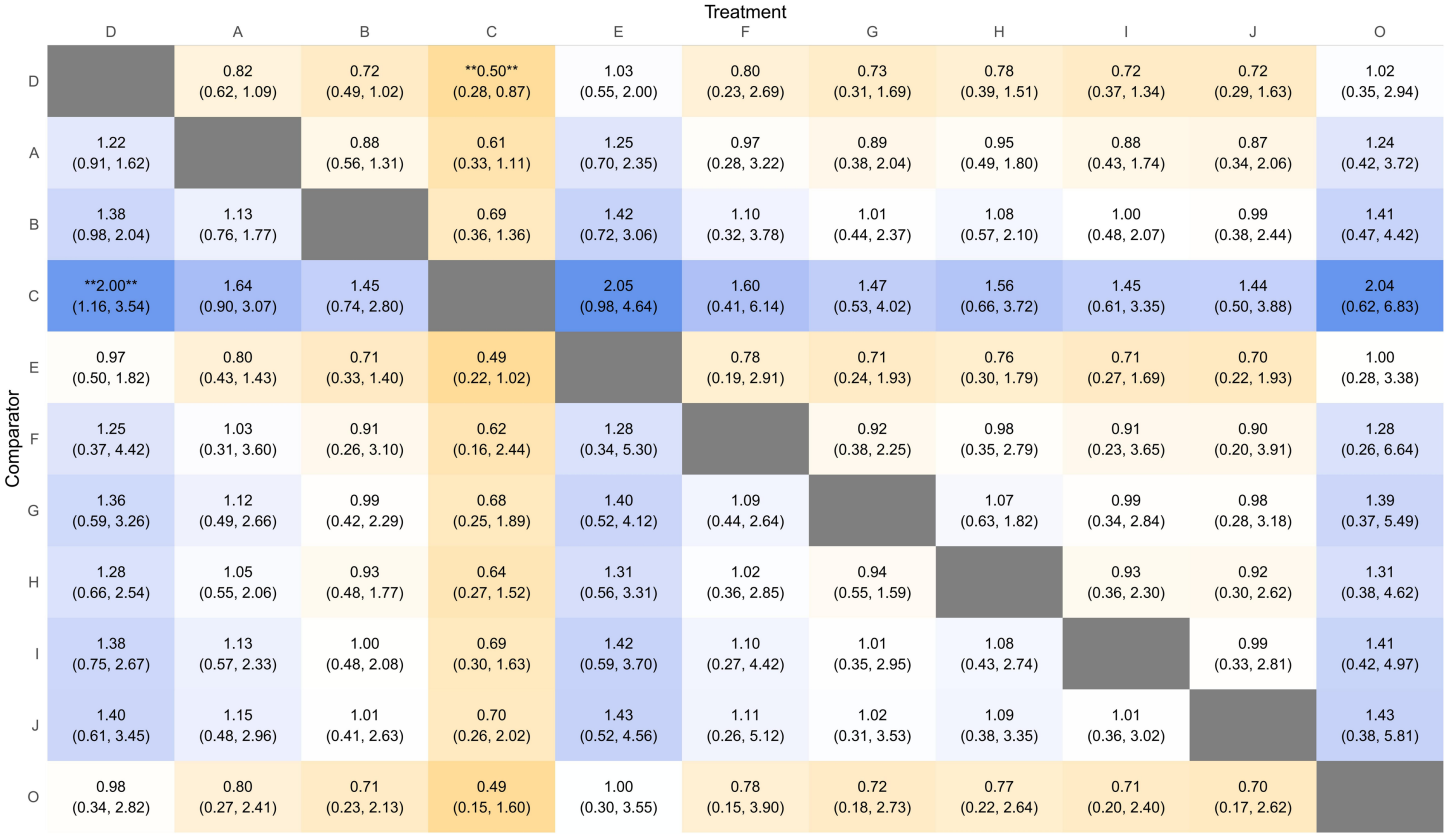
League table of pairwise comparisons for overall perineal laceration. D, Routine care; A, Massage; B, Warm compresses; C, Exercise; E, Education; F, Massage + warm compresses; G, Hands-on techniques; H, Hands-off techniques; I, Lubrication; J, Lubricated massage; O, Massage + exercise.

**Figure 5:**
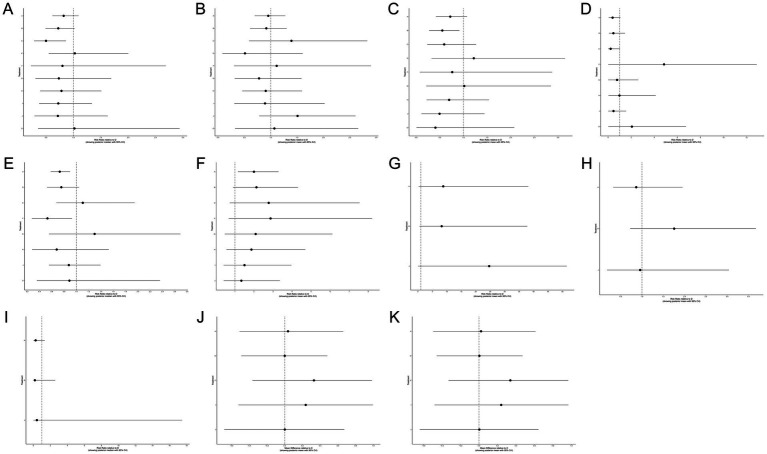
Forest plots comparing physical interventions versus routine care for preventing perineal trauma during vaginal delivery. **(A)** Perineal laceration; **(B)** 1st-degree laceration; **(C)** 2nd-degree laceration; **(D)** Severe laceration (3rd/4th degree); **(E)** Episiotomy; **(F)** Intact perineum; **(G)** Mild pain†; **(H)** Moderate pain†; **(I)** Severe pain†; **(J)** 1-min Apgar score†; **(K)** 5-min Apgar score†. †: tar-shaped structure with routine care as the sole comparator and no direct comparisons between active interventions. Effect estimates relative to routine care are presented; rankings among active interventions are based on indirect evidence only and should be interpreted with caution.

**Figure 6:**
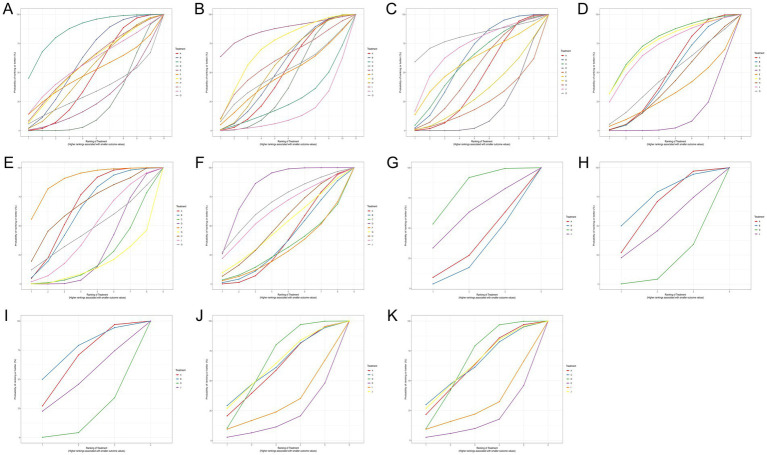
Surface under the cumulative ranking curve (SUCRA) curves for different outcomes of physical interventions for preventing perineal trauma. SUCRA values represent the probability that each intervention is among the best options and should be interpreted as probabilistic summaries rather than definitive rankings. **(A)** Perineal laceration; **(B)** 1st-degree laceration; **(C)** 2nd-degree laceration; **(D)** Severe laceration (3rd/4th degree); **(E)** Episiotomy; **(F)** Intact perineum; **(G)** Mild pain†; **(H)** Moderate pain†; **(I)** Severe pain†; **(J)** 1-min Apgar score†; **(K)** 5-min Apgar score†. †Sparse network (star-shaped): Rankings based on indirect evidence only; confidence in relative rankings is limited.

SUCRA ranking analysis (Figure 3B) suggested that exercise had the highest probability of being the best intervention for overall laceration prevention (SUCRA = 86.58%), followed by warm compresses (SUCRA = 61.02%), lubrication (SUCRA = 58.17%), and hands-on techniques (SUCRA = 57.20%). Education (SUCRA = 26.60%) and routine care (SUCRA = 23.09%) had the lowest probabilities of preventing perineal laceration (Figure 4). For exercise versus routine care, the posterior *τ* = 0.413 (95% CrI: 0.24–0.66; τ^2^ = 0.170) indicates moderate between-study heterogeneity (Supplementary Table S3). Forest plots comparing all physical interventions versus routine care across multiple outcomes are shown in Figure 5. SUCRA curves for all evaluated outcomes are comprehensively presented in Figure 6.

### 3.5 Secondary outcomes

#### 3.5.1 Perineal laceration by severity grade

**Figure 7:**
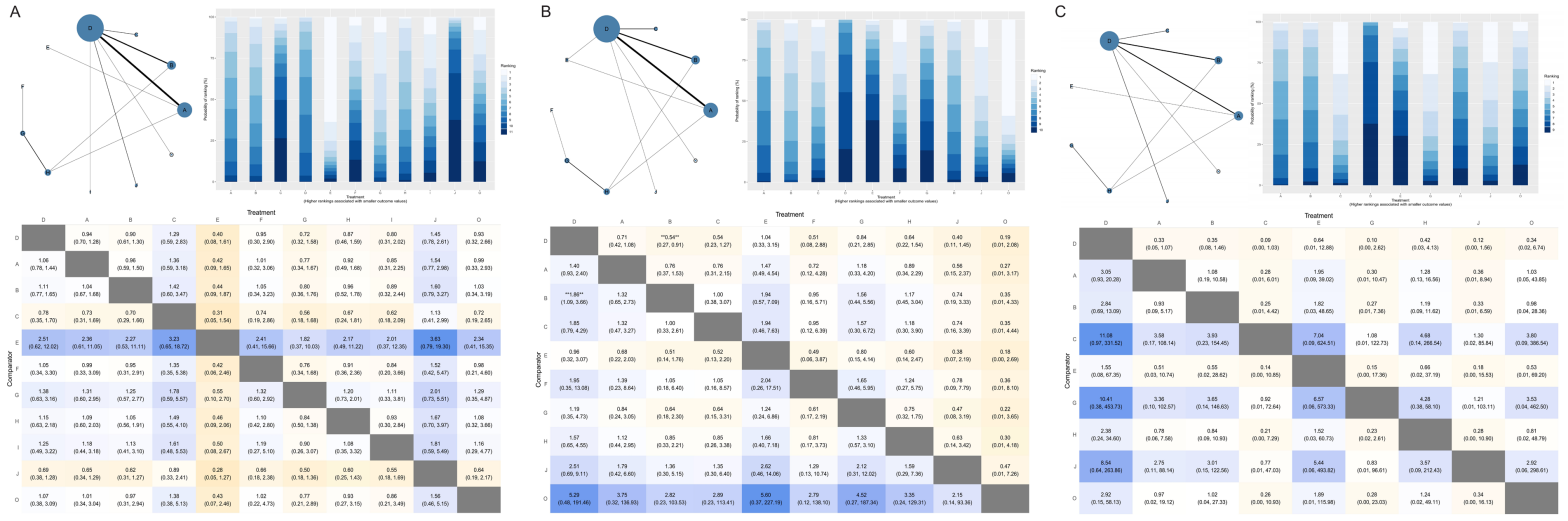
Network meta-analysis results for perineal laceration by severity grade. **(A)** First-degree perineal laceration; **(B)** second-degree perineal laceration; **(C)** severe perineal laceration (third/fourth-degree). D, Routine care; A, Massage; B, Warm compresses; C, Exercise; E, Education; F, Massage + warm compresses; G, Hands-on techniques; H, Hands-off techniques; I, Lubrication; J, Lubricated massage; O, Massage + exercise.

First-degree laceration: The network comprised 11 intervention nodes across 29 RCTs with 9,333 participants, generating 1,908 events (20.4% event rate). No intervention achieved statistical significance compared to routine care. Network geometries for perineal lacerations stratified by severity grade are shown in Figure 7. SUCRA ranking showed education (SUCRA = 86.20%) and hands-on techniques (SUCRA = 71.12%) had the highest probabilities, though extremely wide credible intervals reflected substantial uncertainty (Figure 5). Second-degree laceration: Analysis of 29 RCTs with 8,902 participants (1,635 events, 18.4% event rate) demonstrated that warm compresses significantly reduced second-degree lacerations [RR = 0.54, 95%CrI (0.27, 0.91)]. SUCRA ranking identified massage combined with exercise (SUCRA = 81.20%) and lubricated massage (SUCRA = 71.15%) as top-ranked interventions, followed by warm compresses (SUCRA = 62.26%) (Figure 6). Severe perineal laceration (third/fourth-degree): Analysis of 20 RCTs with 8,184 participants revealed only 302 severe laceration events (3.69% event rate). This network is underpowered to detect statistically significant effects for any intervention,since no intervention achieved statistical significance, all credible intervals are extremely wide and cross the null, and SUCRA rankings for this outcome should be treated as unreliable given the event sparsity. Exercise (SUCRA = 77.23%), hands-on techniques (SUCRA = 75.36%), and lubricated massage (SUCRA = 71.57%) ranked highest. Extremely wide credible intervals necessitate cautious interpretation.

#### 3.5.2 Episiotomy and intact perineum rate

**Figure 8:**
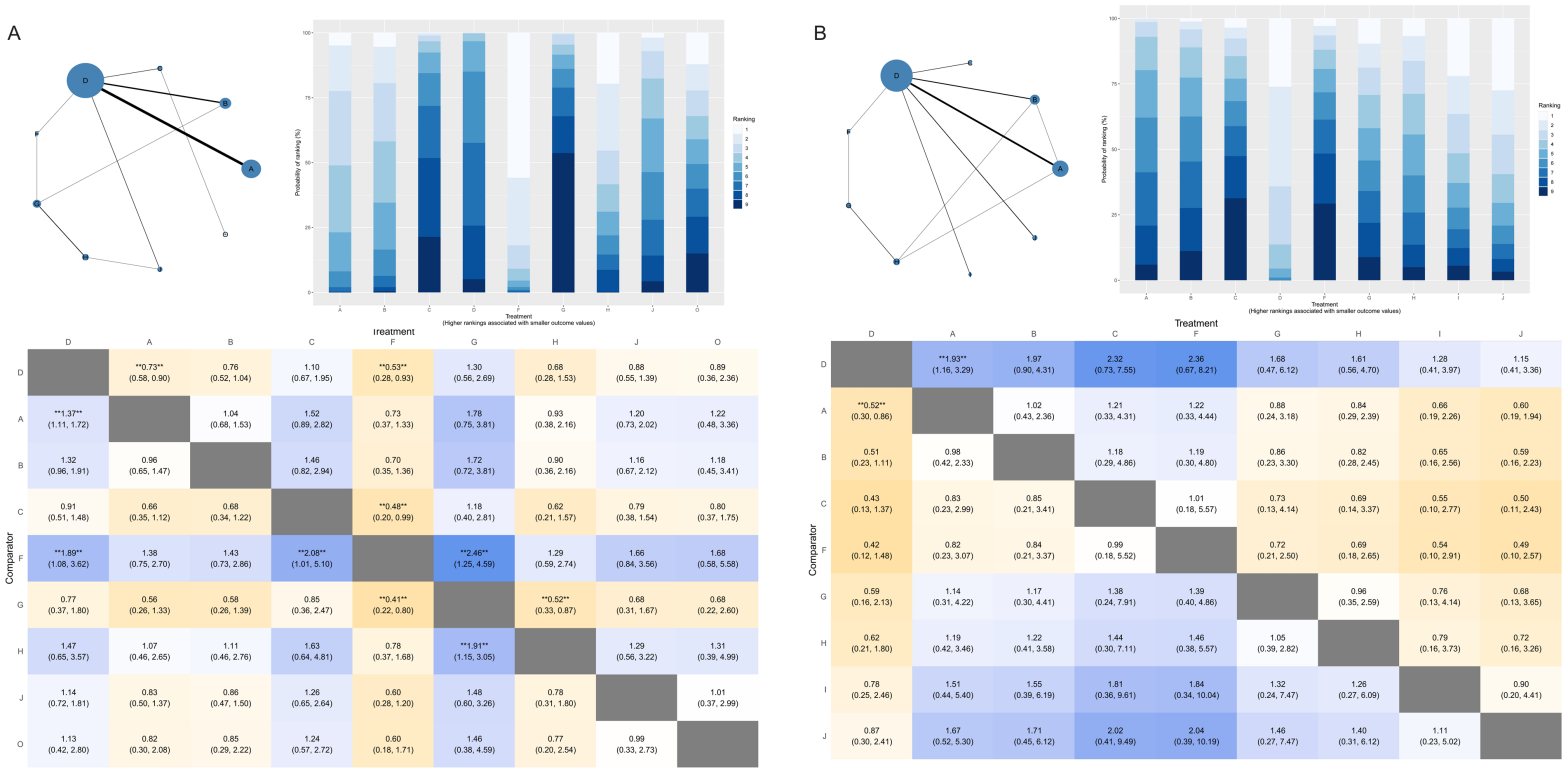
Network meta-analysis results for **(A)** Episiotomy and **(B)** Intact perineum outcomes. D, Routine care; A, Massage; B, Warm compresses; C, Exercise; E, Education; F, Massage + warm compresses; G, Hands-on techniques; H, Hands-off techniques; I, Lubrication; J, Lubricated massage; O, Massage + exercise.

Episiotomy rate: Analysis of 30 RCTs with 8,645 participants (2,431 events, 28.1% event rate) showed massage combined with warm compresses [RR = 0.53, 95%CrI (0.28, 0.93)] and massage alone [RR = 0.73, 95%CrI (0.58, 0.90)] significantly reduced episiotomy rates. SUCRA ranking suggested that massage combined with warm compresses had the highest probability of being the best intervention for episiotomy prevention (SUCRA = 90.08%) (Figure 8A). Intact perineum: Analysis of 26 RCTs with 8,949 participants (2,377 events, 26.6% event rate) demonstrated massage significantly increased intact perineum rates [RR = 1.93, 95%CrI (1.16, 3.29)]. Other interventions including warm compresses [RR = 1.97], exercise [RR = 2.32], and massage combined with warm compresses [RR = 2.36] showed favorable trends (Figure 8B).

#### 3.5.3 Perineal pain

**Figure 9:**
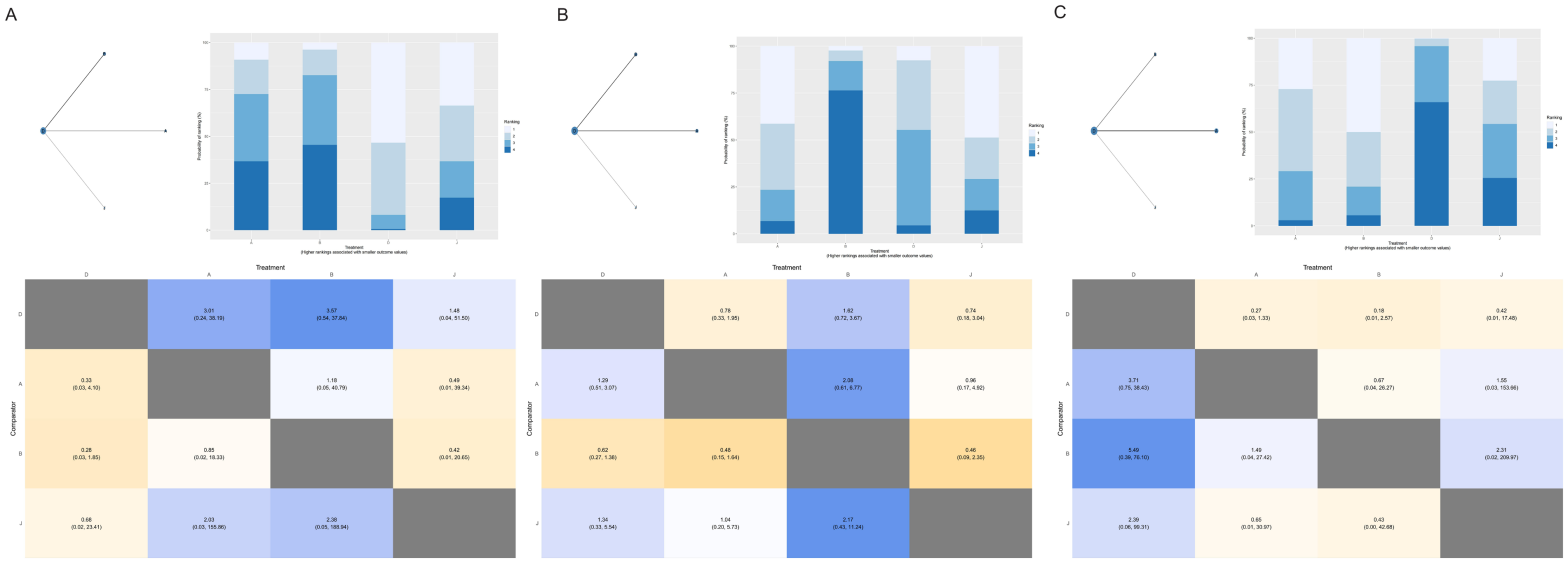
Network meta-analysis results for perineal pain outcomes. All three pain networks **(A–C)** are sparse and star-shaped, with each active intervention connected only to routine care (D) and no direct comparisons between active interventions. **(A)** Mild perineal pain; **(B)** Moderate perineal pain; **(C)** Severe perineal pain. D, Routine care; A, Massage; B, Warm compresses; C, Exercise; E, Education; F, Massage + warm compresses; G, Hands-on techniques; H, Hands-off techniques; I, Lubrication; J, Lubricated massage; O, Massage + exercise.

Pain outcomes were evaluated across mild, moderate, and severe categories. Importantly, the networks for all three pain outcomes were sparse and star-shaped, with each active intervention connected exclusively to routine care and no direct head-to-head comparisons between active interventions available (Figure 9). This configuration means that all relative effect estimates between active interventions are derived entirely from indirect evidence through a single common comparator, which substantially limits the reliability of relative rankings and precludes firm conclusions about comparative efficacy. These findings should therefore be considered exploratory and hypothesis-generating rather than definitive.

##### 3.5.3.1 Mild perineal pain

Analysis of 6 RCTs with 1,604 participants (347 events, 21.6%) showed paradoxical trends with massage [RR = 3.01, 95%CrI (0.24, 38.19)] and warm compresses [RR = 3.57, 95%CrI (0.54, 37.84)] associated with increased mild pain risk, though extremely wide credible intervals reflected profound uncertainty.

##### 3.5.3.2 Moderate perineal pain

Analysis of 7 RCTs with 1,708 participants (413 events, 24.2%) demonstrated massage showed protective trends [RR = 0.78, 95%CrI (0.33, 1.95)], while warm compresses showed increased risk trends [RR = 1.62, 95%CrI (0.72, 3.67)].

##### 3.5.3.3 Severe perineal pain

Analysis of 7 RCTs with 1,703 participants (604 events, 35.5%) revealed substantial protective effects for warm compresses [RR = 0.18, 95%CrI (0.01, 2.57), SUCRA = 74.51%] and massage [RR = 0.27, 95%CrI (0.03, 1.33), SUCRA = 65.01%], representing 82 and 73% risk reductions, respectively.

#### 3.5.4 Neonatal Apgar scores

**Figure 10:**
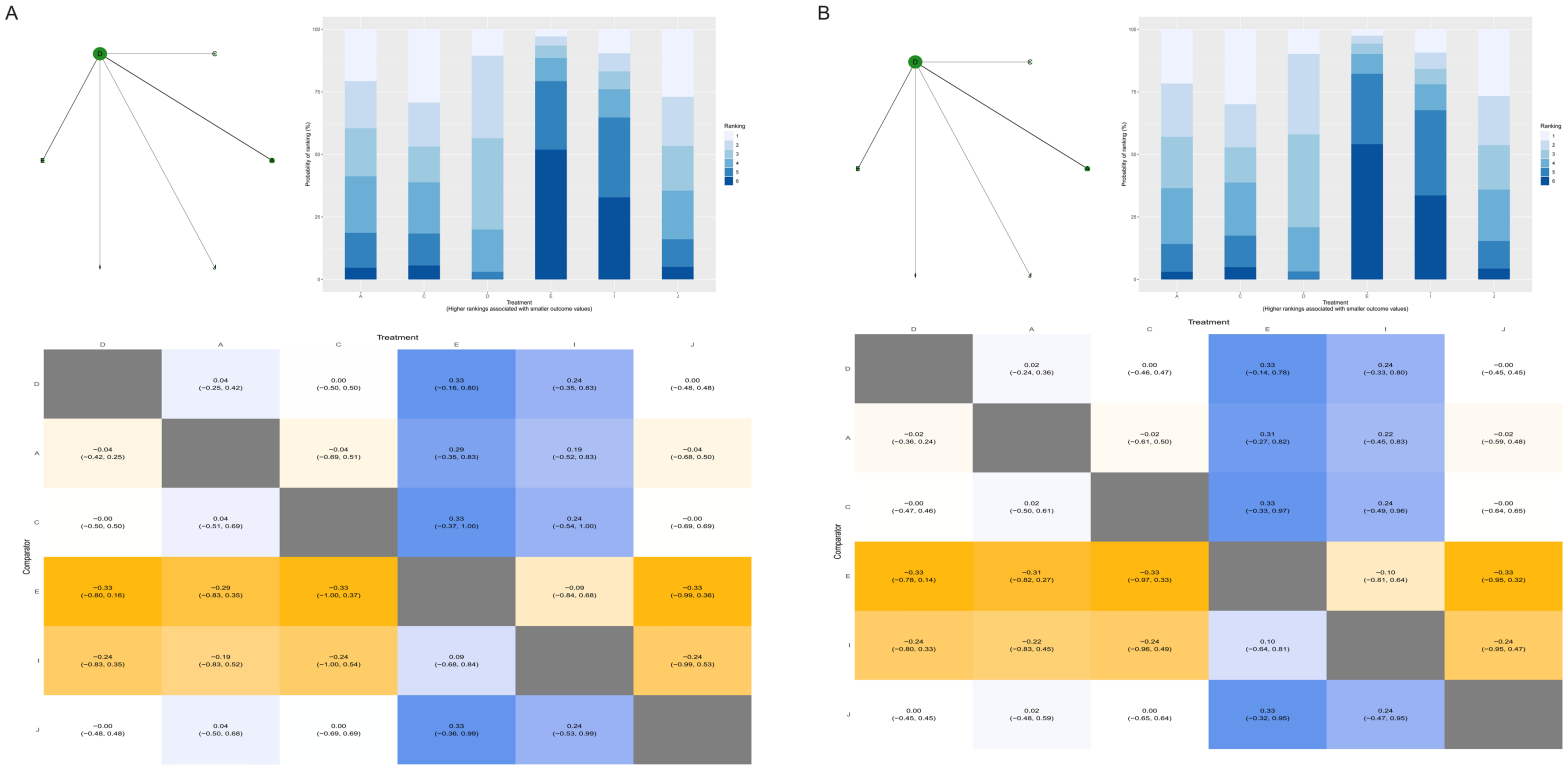
Network meta-analysis results for neonatal Apgar scores. Both networks **(A,B)** are pure star-shaped structures with routine care (D) as the sole central hub and no direct comparisons between active interventions, limiting confidence in relative rankings among active interventions. Effect estimates versus routine care are reliable; however, comparisons between active interventions are based entirely on indirect evidence. **(A)** Apgar score at 1 min; **(B)** Apgar score at 5 min. D, Routine care; A, Massage; B, Warm compresses; C, Exercise; E, Education; F, Massage + warm compresses; G, Hands-on techniques; H, Hands-off techniques; I, Lubrication; J, Lubricated massage; O, Massage + exercise.

Both 1-min and 5-min Apgar scores were evaluated across 8 RCTs with approximately 2,200 participants. The networks demonstrated pure star-shaped structures with routine care as the central hub and no direct comparisons between active interventions (Figure 10). As with the pain outcomes, this sparse configuration means that rankings among active interventions are based entirely on indirect evidence, limiting confidence in relative comparisons between them. Nonetheless, the consistent pattern across all interventions permits reliable assessment of each intervention’s effect relative to routine care.

All physical interventions showed negligible effects on both 1-min and 5-min Apgar scores compared to routine care, with all mean differences within ±0.33 points—well below the ≥1 point threshold for clinical significance. These findings provide strong reassurance regarding neonatal safety across all evaluated interventions, indicating that optimizing maternal perineal outcomes through physical interventions does not compromise neonatal wellbeing.

## 4 Discussion

This Bayesian network meta-analysis synthesizing 31 randomized controlled trials with 10,745 participants represents the first comprehensive comparison of all available physical interventions for perineal protection during vaginal delivery (29). Network meta-analysis overcomes the limitations of traditional pairwise meta-analyses by integrating both direct and indirect evidence within a unified analytical framework, enabling comprehensive evaluation and probabilistic ranking across all physical approaches. This integrated approach provides evidence-based clinical guidance for selecting optimal strategies in contemporary obstetric practice (30, 31).

This NMA directly demonstrated that antenatal pelvic floor exercise produced the most robust protective effect against overall perineal laceration (RR = 0.50, 95%CrI 0.28–0.87), while intrapartum massage significantly reduced episiotomy rates (RR = 0.73, 95%CrI 0.58–0.90) and promoted intact perineum (RR = 1.93, 95%CrI 1.16–3.29), and warm compresses significantly reduced second-degree lacerations (RR = 0.54, 95%CrI 0.27–0.91). These distinct outcome profiles suggest that antenatal conditioning and intrapartum physical interventions operate through fundamentally different protective mechanisms, which may explain why their efficacy profiles differ across laceration severity grades and clinical outcomes (32, 33). The superior protective effect of antenatal exercise is consistent with evidence from external literature indicating that sustained pelvic floor muscle training over weeks to months enhances muscle fiber elasticity, improves neuromuscular coordination, and promotes organized collagen remodeling (34, 35). During pregnancy, relaxin-mediated hormonal changes progressively reduce pelvic floor tissue stiffness by inhibiting collagen synthesis and increasing tissue compliance (36, 37); structured exercise is proposed to partially counteract these changes by preserving tissue architecture and improving the capacity of pelvic floor muscles to accommodate the extreme mechanical demands of the second stage of labor (38, 39). For intrapartum interventions, warm compresses are thought to act through local vasodilation and thermally-induced reductions in collagen stiffness, increasing tissue compliance and extensibility during fetal descent (40–42), while perineal massage is proposed to promote acute muscle relaxation and facilitate gradual tissue distension, thereby lowering the clinical threshold for episiotomy and reducing the abrupt mechanical loading that contributes to severe lacerations (43–45). These proposed mechanisms are supported by external biomechanical and physiological studies. However, the precise pathways by which each intervention confers perineal protection have not been directly tested within the framework of the current NMA, and should be regarded as the most plausible current explanations rather than established facts.

The observation that massage was associated with trends toward increased first-degree lacerations while simultaneously reducing episiotomy rates and severe trauma raises the speculative hypothesis that controlled, superficial tissue disruption may dissipate mechanical forces that would otherwise propagate into deeper tissue planes, thereby preventing more clinically significant injuries (46–48). This interpretation is consistent with the aggregate pattern of NMA findings but cannot be confirmed from trial-level data alone, and requires prospective validation through biomechanical studies or individual patient data analyses. Similarly, the apparent pattern in pain outcomes that with trends toward increased mild pain alongside reduced severe pain for both massage and warm compresses (49) could speculatively suggest that these interventions shift the distribution of perineal pain toward less severe grades; however, as discussed above, this observation is not supported by statistically significant estimates and should be treated as a hypothesis for future investigation rather than a clinical conclusion (50). Whether the overall outcome profile associated with massage and warm compresses represents a net benefit acceptable to women warrants dedicated patient-centered research incorporating validated quality of life and pain endpoints.

Our analysis found no statistically significant differences between hands-on (RR = 0.73) and hands-off (RR = 0.78) approaches for overall laceration prevention, though both showed protective trends compared to routine care (51, 52). This clinical equipoise reflects ongoing controversy regarding optimal perineal management during crowning. The similar efficacy of both approaches suggests that skilled clinical judgment and adaptability to individual labor dynamics may be more important than adherence to a specific protocol, with experienced practitioners achieving comparable outcomes regardless of technique (53). The lack of clear superiority indicates both represent acceptable clinical strategies, with selection appropriately guided by provider experience, clinical circumstances, maternal tissue characteristics, and maternal preferences (54).

Education interventions without accompanying physical training showed no protective effects (RR = 1.03, SUCRA = 26.60%), emphasizing the necessity of active biomechanical interventions rather than passive knowledge transfer (55). The lack of efficacy highlights a critical distinction between understanding perineal protection principles and implementing effective behavioral modifications, underscoring the importance of active interventions that directly modify tissue biomechanics. Women receiving education alone likely understand prevention concepts but cannot independently execute effective perineal protection strategies without physical training programs that build muscle capacity and tissue resilience or skilled provider application of intrapartum techniques during delivery (56).

The absence of clinically meaningful effects on Apgar scores at both 1 and 5 min (all mean differences <0.33 points, well below the ≥1.0 threshold for clinical significance) provides strong reassurance regarding neonatal safety across all evaluated interventions (57). Physical interventions for maternal perineal protection do not compromise fetal oxygenation, acid–base status, or cardiovascular adaptation during delivery. The high-quality evidence supporting neonatal safety enables clinicians to focus decision-making exclusively on maternal benefits without concerns about fetal compromise, representing a critical advantage of physical interventions (58).

Based on our analysis, we suggest a hierarchical framework to guide evidence-informed clinical decision-making for perineal protection during vaginal delivery. Antenatal pelvic floor exercise programs represent a promising first-line prevention strategy for primiparous women beginning in the second trimester,supported by a statistically significant 50% reduction in overall perineal trauma and the highest SUCRA probability ranking (33, 59). For women presenting in labor without prior pelvic floor training, warm compresses applied during the second stage represent an evidence-informed intrapartum option, supported by significant reduction in second-degree lacerations (RR = 0.54, 95%CrI 0.27–0.91; moderate-certainty evidence) and a favorable SUCRA profile for severe pain reduction, though pain outcome data remain exploratory given sparse network structures and wide credible intervals. Intrapartum perineal massage offers an alternative supported by significant episiotomy reduction (RR = 0.73, 95%CrI 0.58–0.90; moderate-certainty evidence) and significant promotion of intact perineum (RR = 1.93, 95%CrI 1.16–3.29; moderate-certainty evidence) (49, 60). Hands-on and hands-off perineal management techniques both showed protective trends without significant differences between them (low-certainty evidence), and selection should be guided by clinical circumstances, maternal preferences, and provider experience (51, 52). However, our study has important limitations. Firstly, qualitative consideration of publication bias risk is important in this literature, everal features suggest potential susceptibility, including the predominance of single-centre trials from academic settings, the absence of confirmed null-result publications for several intervention types in trial registries, and the concentration of positive findings in specific intervention categories. Meanwhile, massage as the most frequently studied comparison yet substantially more positive results than neutral ones,which raises the possibility of selective reporting. Therefore, publication bias towards positive results cannot be excluded, and this uncertainty is reflected in the GRADE certainty ratings for relevant outcomes. Second, the sparse, star-shaped networks for pain and Apgar outcomes mean that relative rankings among active interventions for these outcomes are derived entirely from indirect evidence and should be considered exploratory. For exercise versus routine care, the 95% predictive interval indicates that, while the summary estimate reflects a substantial protective effect, the true effect in a new clinical setting could range considerably, reflecting the between-study variance inherent in this network. This underscores the importance of contextual factors in determining intervention effectiveness and should temper the strength of universal clinical recommendations. Extreme event sparsity for severe third/fourth-degree lacerations produced wide credible intervals precluding definitive statistical conclusions. Substantial heterogeneity in intervention protocols prevents identification of optimal implementation parameters (61). The evidence base of this NMA applies primarily to primiparous women with term singleton pregnancies, as 80.6% of included trials exclusively enrolled this population. Meanwhile, included trials showed substantial clinical heterogeneity in intervention protocols, operator training, and study populations. A DIC difference indicates meaningful variation in true effects across settings, a primiparous-specific sensitivity analysis therefore was not feasible. SUCRA rankings should therefore be interpreted as probabilistic estimates rather than precise rankings universally applicable across clinical contexts. Generalizability to multiparous women who have different baseline perineal tissue characteristics, shorter second stages of labor, and lower baseline trauma rates remains uncertain and cannot be directly inferred from the current evidence. Furthermore, the majority of included trials were conducted in high-resource settings with standardized obstetric care, access to trained providers, and consistent intervention delivery. The applicability of these findings to low- or middle-income countries, where resource availability, clinical staffing, episiotomy rates, and obstetric practices differ substantially, is therefore uncertain and warrants dedicated investigation in these contexts (62). Short-term follow-up precludes assessment of long-term outcomes including sexual function recovery and development of pelvic floor disorders (6).

Despite these limitations, our network meta-analysis provides the most comprehensive synthesis currently available of physical interventions for perineal protection, offering probabilistic hierarchical rankings intended to guide rather than prescribe clinical decision-making. Antenatal pelvic floor exercise is identified as a promising first-line strategy supported by moderate-certainty evidence, with warm compresses and massage representing evidence-informed intrapartum alternatives. Broader implementation and rigorous prospective evaluation of these strategies, particularly among underrepresented populations and in varied clinical settings, could contribute to reducing the burden of perineal trauma and improving maternal outcomes (63, 64).

## 5 Conclusion

This Bayesian network meta-analysis of 31 randomized controlled trials involving 10,745 participants across 15 countries represents the first systematic comparison of all available physical interventions for perineal protection during vaginal delivery. Antenatal pelvic floor exercise demonstrated the highest probability of being the most effective intervention for overall perineal laceration prevention, and represents a promising first-line strategy for primiparous women in well-resourced settings. Warm compresses and perineal massage represent evidence-informed intrapartum alternatives supported by moderate-certainty evidence for specific outcomes including second-degree laceration prevention and episiotomy reduction, respectively. All physical interventions demonstrated excellent neonatal safety. Based on these findings, we suggest broader clinical evaluation and implementation of antenatal pelvic floor exercise programs beginning in the second trimester, with warm compresses and perineal massage as evidence-informed intrapartum options for women without prior training. These strategies hold potential to reduce the burden of perineal trauma; however, further high-quality research in diverse populations and clinical settings is needed before broader generalizations can be made. Moreover, further high-quality trials with standardized protocols, long-term follow-up assessing sexual function and quality of life are needed to refine these recommendations and identify optimal implementation parameters for clinical practice.

## Data Availability

The original contributions presented in the study are included in the article/Supplementary material, further inquiries can be directed to the corresponding authors.
