# Supplementary figures and images for: Comparative effectiveness of physical interventions for preventing perineal trauma during vaginal delivery: a systematic review and Bayesian network meta-analysis
Source: Front Med (Lausanne). 2026 Apr 7;13:1794056. doi: 10.3389/fmed.2026.1794056 (PMC13096049; doi:10.3389/fmed.2026.1794056)

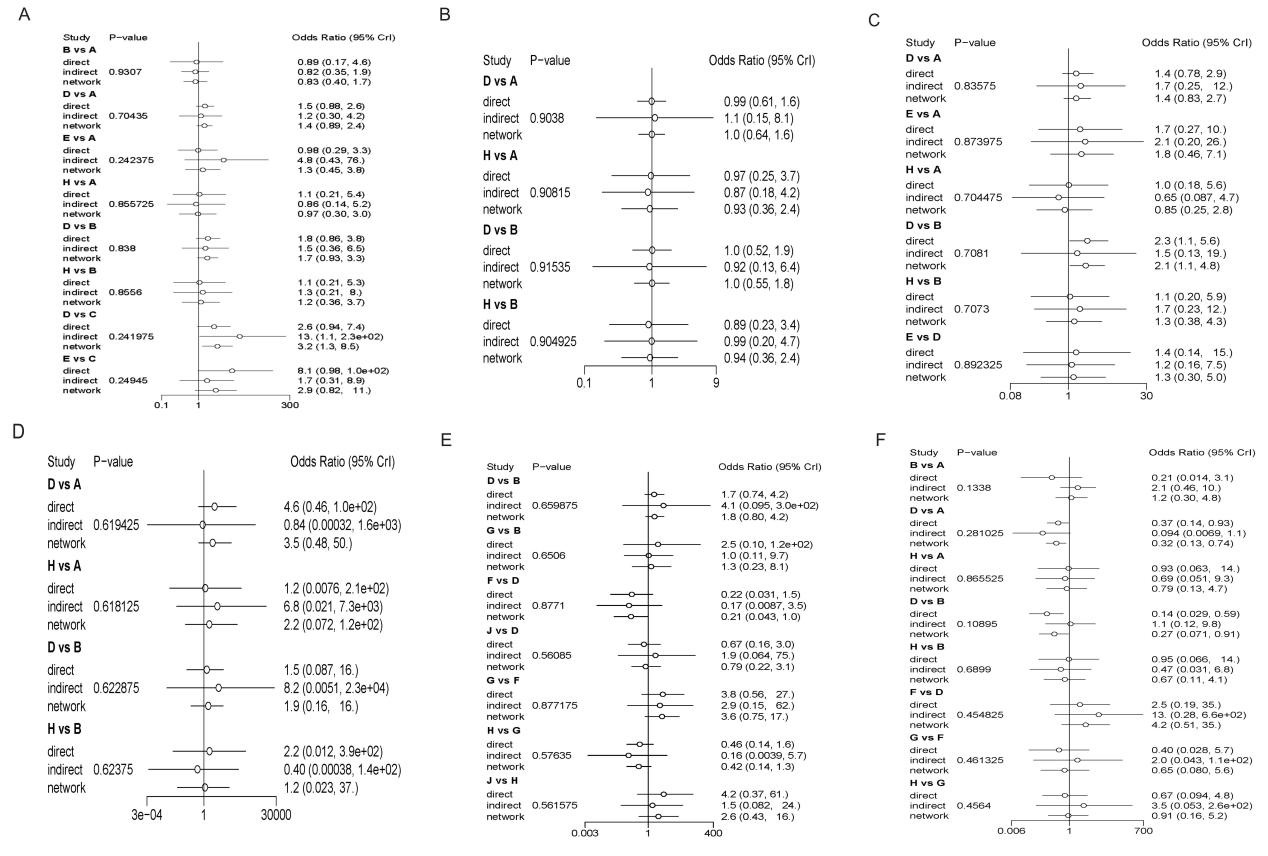

Supplement: Supplementary file 1 [file Image_1.jpg]

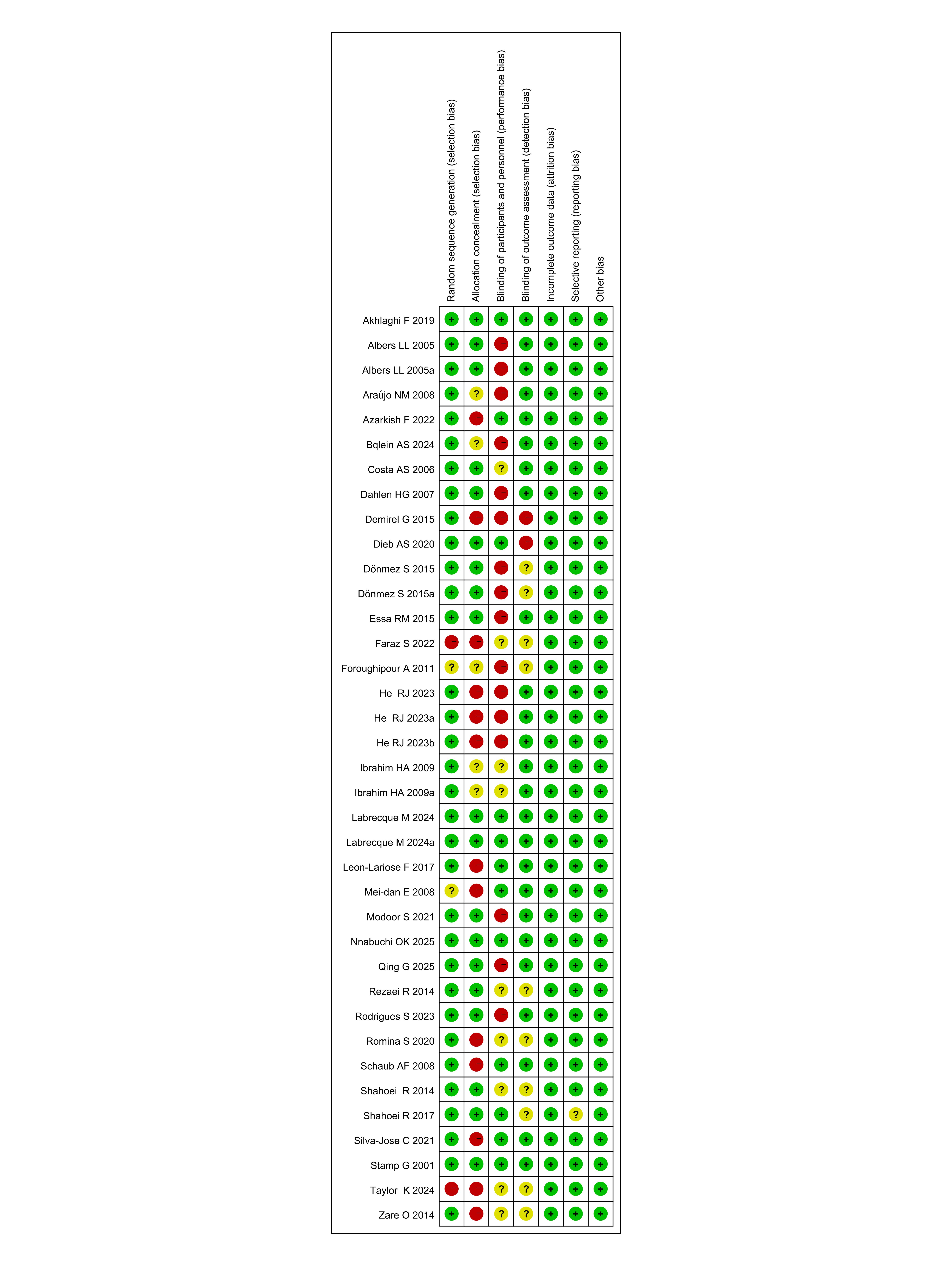

Supplement: Supplementary file 2 [file Image_2.jpg]
