# Supplementary material for: Comparative effectiveness of physical interventions for preventing perineal trauma during vaginal delivery: a systematic review and Bayesian network meta-analysis
Source: Front Med (Lausanne). 2026 Apr 7;13:1794056. doi: 10.3389/fmed.2026.1794056 (PMC13096049; doi:10.3389/fmed.2026.1794056)
Supplement: Supplementary file 3 [file Table_1.docx]

Supplementary Table S1. Complete Search Strategies for All Four Databases

Database 1: PubMed

| Number | Search terms |
| --- | --- |
| #1 | "Perineal Injuries"[Mesh] OR "perineal injury"[Title/Abstract] OR "perineal damage"[Title/Abstract] OR "perineal trauma"[Title/Abstract] OR "perineal tear"[Title/Abstract] OR "perineal laceration"[Title/Abstract] OR "perineal rupture"[Title/Abstract] OR "perineal wound"[Title/Abstract] OR "obstetric trauma"[Title/Abstract] |
| #2 | "Labor, Obstetric"[Mesh] OR "Delivery, Obstetric"[Mesh] OR "vaginal delivery"[Title/Abstract] OR "vaginal birth"[Title/Abstract] OR "childbirth"[Title/Abstract] OR "labor"[Title/Abstract] OR "labour"[Title/Abstract] OR "parturition"[Title/Abstract] OR "second stage"[Title/Abstract] |
| #3 | "Massage"[Mesh] OR "perineal massage"[Title/Abstract] OR "warm compress"[Title/Abstract] OR "hot compress"[Title/Abstract] OR "perineal compress"[Title/Abstract] OR "Exercise"[Mesh] OR "pelvic floor exercise"[Title/Abstract] OR "pelvic floor muscle training"[Title/Abstract] OR "Kegel exercise"[Title/Abstract] OR "hands-on technique"[Title/Abstract] OR "hands-off technique"[Title/Abstract] OR "perineal support"[Title/Abstract] OR "lubrication"[Title/Abstract] OR "perineal oil"[Title/Abstract] OR "Patient Education"[Mesh] OR "prenatal education"[Title/Abstract] OR "antenatal education"[Title/Abstract] OR "physical intervention"[Title/Abstract] OR "perineal protection"[Title/Abstract] OR "perineal management"[Title/Abstract] |
| #4 | "Randomized Controlled Trial"[Publication Type] OR "Controlled Clinical Trial"[Publication Type] OR "randomized"[Title/Abstract] OR "randomised"[Title/Abstract] OR "randomly"[Title/Abstract] OR "random allocation"[Title/Abstract] OR "clinical trial"[Title/Abstract] OR "RCT"[Title/Abstract] |
| #5 | #1 AND #2 AND #3 AND #4 |
| #6 | Filters: Publication date from 2001/01/01 to 2025/11/30 |

Database 2: Web of Science (Core Collection)

| Number | Search Terms |
| --- | --- |
| #1 | TS=("perineal injury" OR "perineal damage" OR "perineal trauma" OR "perineal tear" OR "perineal laceration" OR "perineal rupture" OR "perineal wound" OR "obstetric perineal trauma" OR "obstetric anal sphincter" OR "OASIS" OR "perineal lacerations") |
| #2 | TS=("vaginal delivery" OR "vaginal birth" OR "childbirth" OR "vaginal labour" OR "vaginal labor" OR "second stage of labor" OR "second stage of labour" OR "parturition" OR "obstetric delivery") |
| #3 | TS=("perineal massage" OR "intrapartum massage" OR "antenatal massage" OR "warm compress" OR "hot compress" OR "perineal compress" OR "heat application" OR "pelvic floor exercise" OR "pelvic floor muscle training" OR "pelvic floor muscle exercise" OR "Kegel exercise" OR "antenatal exercise" OR "prenatal exercise" OR "hands-on technique" OR "hands-off technique" OR "hands on" OR "hands off" OR "manual perineal support" OR "perineal support" OR "controlled fetal head delivery" OR "lubrication" OR "perineal oil" OR "lubricant gel" OR "prenatal education" OR "antenatal education" OR "childbirth education" OR "physical intervention" OR "perineal protection" OR "perineal management") |
| #4 | TS=("randomized controlled trial" OR "randomised controlled trial" OR "controlled clinical trial" OR "randomized" OR "randomised" OR "randomly allocated" OR "random allocation" OR "RCT" OR "clinical trial") |
| #5 | #1 AND #2 AND #3 AND #4 |
| #6 | Refined by: Document Types: Article OR Review; Timespan: 2001-01-01 to 2025-11-30 |

Database 3: Embase (via Elsevier)

Search period: January 1, 2001 – November 30, 2025

| Number | Search Terms |
| --- | --- |
| #1 | 'perineum injury'/exp OR 'perineal injury':ab,ti OR 'perineal damage':ab,ti OR 'perineal trauma':ab,ti OR 'perineal tear':ab,ti OR 'perineal laceration':ab,ti OR 'perineal rupture':ab,ti OR 'perineal wound':ab,ti OR 'obstetric perineal trauma':ab,ti OR 'obstetric anal sphincter injury':ab,ti OR 'OASIS':ab,ti OR 'perineal lacerations':ab,ti |
| #2 | 'vaginal delivery'/exp OR 'obstetric delivery'/exp OR 'vaginal delivery':ab,ti OR 'vaginal birth':ab,ti OR 'vaginal labour':ab,ti OR 'vaginal labor':ab,ti OR 'childbirth':ab,ti OR 'second stage of labor':ab,ti OR 'second stage of labour':ab,ti OR 'parturition':ab,ti |
| #3 | 'massage'/exp OR 'perineal massage':ab,ti OR 'intrapartum massage':ab,ti OR 'antenatal massage':ab,ti OR 'warm compress':ab,ti OR 'hot compress':ab,ti OR 'perineal compress':ab,ti OR 'heat application':ab,ti OR 'pelvic floor exercise'/exp OR 'pelvic floor muscle training':ab,ti OR 'pelvic floor exercise':ab,ti OR 'pelvic floor muscle exercise':ab,ti OR 'Kegel exercise':ab,ti OR 'antenatal exercise':ab,ti OR 'prenatal exercise':ab,ti OR 'hands-on technique':ab,ti OR 'hands-off technique':ab,ti OR 'manual perineal support':ab,ti OR 'perineal support':ab,ti OR 'controlled fetal head delivery':ab,ti OR 'lubrication':ab,ti OR 'perineal oil':ab,ti OR 'lubricant gel':ab,ti OR 'patient education'/exp OR 'prenatal education':ab,ti OR 'antenatal education':ab,ti OR 'childbirth education':ab,ti OR 'physical intervention':ab,ti OR 'perineal protection':ab,ti OR 'perineal management':ab,ti |
| #4 | 'randomized controlled trial'/exp OR 'controlled clinical trial'/exp OR 'randomized':ab,ti OR 'randomised':ab,ti OR 'randomly':ab,ti OR 'random allocation':ab,ti OR 'RCT':ab,ti OR 'clinical trial':ab,ti |
| #5 | #1 AND #2 AND #3 AND #4 |
| #6 | Limit to: Article; Publication year: 2001–2025 |

Database 4: Cochrane Central Register of Controlled Trials (CENTRAL)

Search period: January 1, 2001 – November 30, 2025

| Number | Search Terms |
| --- | --- |
| #1 | MeSH descriptor: [Perineal Injuries] explode all trees OR "perineal injury" OR "perineal damage" OR "perineal trauma" OR "perineal tear" OR "perineal laceration" OR "perineal rupture" OR "perineal wound" OR "obstetric perineal trauma" OR "obstetric anal sphincter" |
| #2 | MeSH descriptor: [Labor, Obstetric] explode all trees OR MeSH descriptor: [Delivery, Obstetric] explode all trees OR "vaginal delivery" OR "vaginal birth" OR "childbirth" OR "second stage of labor" OR "second stage of labour" OR "parturition" |
| #3 | MeSH descriptor: [Massage] explode all trees OR MeSH descriptor: [Exercise] explode all trees OR MeSH descriptor: [Patient Education as Topic] explode all trees OR "perineal massage" OR "warm compress" OR "hot compress" OR "perineal compress" OR "pelvic floor exercise" OR "pelvic floor muscle training" OR "Kegel exercise" OR "hands-on technique" OR "hands-off technique" OR "manual perineal support" OR "perineal support" OR "lubrication" OR "perineal oil" OR "prenatal education" OR "antenatal education" OR "physical intervention" OR "perineal protection" OR "perineal management" |
| #4 | #1 AND #2 AND #3 |
| #5 | Limit: Publication Year from 2001 to 2025 |

Note: All searches were conducted without language restrictions. TS = Topic Search (title, abstract, author keywords); ab,ti = abstract and title fields; MeSH = Medical Subject Headings. The search strategies were independently verified by two reviewers (L.X. and T.L.) prior to execution. Any discrepancies in retrieved records were resolved by consensus. The complete search was last updated on November 30, 2025.
