# Supplementary material for: Comparative effectiveness of physical interventions for preventing perineal trauma during vaginal delivery: a systematic review and Bayesian network meta-analysis
Source: Front Med (Lausanne). 2026 Apr 7;13:1794056. doi: 10.3389/fmed.2026.1794056 (PMC13096049; doi:10.3389/fmed.2026.1794056)
Supplement: Supplementary file 4 [file Table_2.pdf]

PRISMA 2020 Checklist

Section and Topic	Item #	Checklist item	Location where item is reported
**TITLE**			
Title	1	Identify the report as a systematic review.	Title (Lines 1–3): "A Systematic Review and Bayesian Network Meta-Analysis"
**ABSTRACT**			
Abstract	2	See the PRISMA 2020 for Abstracts checklist.	Abstract section (Lines 28–66)
**INTRODUCTION**			
Rationale	3	Describe the rationale for the review in the context of existing knowledge.	Introduction, paragraphs 1–3 (Lines 69–116)
Objectives	4	Provide an explicit statement of the objective(s) or question(s) the review addresses.	Introduction, paragraph 4 (Lines 117–134): "we conducted this comprehensive Bayesian network meta-analysis to systematically compare the efficacy and safety of all available physical interventions…"
**METHODS**			
Eligibility criteria	5	Specify the inclusion and exclusion criteria for the review and how studies were grouped for the syntheses.	Methods, Section 2.3 (Lines 160–188)
Information sources	6	Specify all databases, registers, websites, organisations, reference lists and other sources searched or consulted to identify studies. Specify the date when each source was last searched or consulted.	Methods, Section 2.2 (Lines 147–159): "searched four electronic databases: PubMed, Web of Science, Embase, and the Cochrane Central Register of Controlled Trials (CENTRAL) from January 1, 2001, to November 30, 2025"; reference lists also manually searched
Search strategy	7	Present the full search strategies for all databases, registers and websites, including any filters and limits used.	Methods, Section 2.2 (Lines 147–159) and Supplementary Table S1
Selection process	8	Specify the methods used to decide whether a study met the inclusion criteria of the review, including how many reviewers screened each record and each report retrieved, whether they worked independently, and if applicable, details of automation tools used in the process.	Methods, Section 2.3 (Lines 184–188): "two authors independently screened the titles and abstracts…inconsistencies resolved through discussion or consultation with a third reviewer"
Data collection process	9	Specify the methods used to collect data from reports, including how many reviewers collected data from each report, whether they worked independently, any processes for obtaining or confirming data from study investigators, and if applicable, details of automation tools used in the process.	Methods, Section 2.4 (Lines 189–207)
Data items	10a	List and define all outcomes for which data were sought. Specify whether all results that were compatible with each outcome domain in each study were sought (e.g. for all measures, time points, analyses), and if not, the methods used to decide which results to collect.	Methods, Section 2.3 (Lines 173–176): "Primary outcome was overall perineal laceration rate. Secondary outcomes included laceration severity grades, episiotomy rate, intact perineum rate, perineal pain (mild, moderate, or severe), and neonatal outcomes (Apgar scores at 1 and 5 minutes)"
	10b	List and define all other variables for which data were sought (e.g. participant and intervention characteristics,	Methods, Section 2.4 (Lines 195–207):

Section and Topic	Item #	Checklist item	Location where item is reported
		funding sources). Describe any assumptions made about any missing or unclear information.	"extracted information included basic publication information, participant characteristics, intervention details (type, timing, duration, frequency, technique description, comparator, and co-interventions), and outcome data"
Study risk of bias assessment	11	Specify the methods used to assess risk of bias in the included studies, including details of the tool(s) used, how many reviewers assessed each study and whether they worked independently, and if applicable, details of automation tools used in the process.	Methods, Section 2.4 (Lines 208–219): "assessed by two independent reviewers using the Cochrane Risk of Bias tool version 2.0 (RoB 2), which evaluated bias arising from the randomization process, deviations from intended interventions, missing outcome data, outcome measurement, and selection of reported results"
Effect measures	12	Specify for each outcome the effect measure(s) (e.g. risk ratio, mean difference) used in the synthesis or presentation of results.	Methods, Section 2.5 (Lines 225–228): "For dichotomous outcomes, relative risks (RR) with 95% credible intervals (CrI) were calculated; for continuous outcomes, mean differences (MD) with 95% CrIs were computed"
Synthesis methods	13a	Describe the processes used to decide which studies were eligible for each synthesis (e.g. tabulating the study intervention characteristics and comparing against the planned groups for each synthesis (item #5)).	Methods, Sections 2.3 (Lines 160–188) and 2.5 (Lines 220–240)
	13b	Describe any methods required to prepare the data for presentation or synthesis, such as handling of missing summary statistics, or data conversions.	Methods, Section 2.5 (Line 239): "Zero-event arms received 0.5 continuity corrections"
	13c	Describe any methods used to tabulate or visually display results of individual studies and syntheses.	Methods, Section 2.5 (Lines 229–236): "Network diagrams visualized evidence structure, with node sizes reflecting participant numbers and edge thickness indicating study counts…SUCRA curves visualized probability rankings across interventions"
	13d	Describe any methods used to synthesize results and provide a rationale for the choice(s). If meta-analysis was performed, describe the model(s), method(s) to identify the presence and extent of statistical heterogeneity, and software package(s) used.	Methods, Section 2.5 (Lines 221–236): "Bayesian network meta-analyses were performed using R Studio with the gemtc and BUGSnet packages. Both fixed-effects and random-effects models were fitted, with model selection based on deviance information criterion (DIC)… MCMC simulations employed three chains with 50,000 iterations after 20,000 burn-in iterations"
	13e	Describe any methods used to explore possible causes of heterogeneity among study results (e.g. subgroup	Methods, Section 2.5 (Lines 230–234): "Inconsistency between direct and indirect

Section and Topic	Item #	Checklist item	Location where item is reported
		analysis, meta-regression).	evidence was evaluated by comparing consistency versus inconsistency models…node-splitting analysis assessed loop-specific inconsistency (P>0.05 indicating consistency)"
	13f	Describe any sensitivity analyses conducted to assess robustness of the synthesized results.	Methods, Section 2.5 (Lines 230–233); Results, Section 3.2 (Lines 288–294): narrative sensitivity analysis by excluding studies with 'some concerns' in risk of bias assessment
Reporting bias assessment	14	Describe any methods used to assess risk of bias due to missing results in a synthesis (arising from reporting biases).	Results, Section 3.3 (Lines 300–304): "comparison-adjusted funnel plots could not be reliably constructed for most comparisons due to the insufficient number of studies per direct treatment pair (fewer than 10 in most cases)"
Certainty assessment	15	Describe any methods used to assess certainty (or confidence) in the body of evidence for an outcome.	Methods, Section 2.4 (Lines 213–219): "The certainty of evidence for each outcome was assessed using the GRADE approach for network meta-analysis… performed in Review Manager 5.4 and GRADEpro GDT software"
**RESULTS**			
Study selection	16a	Describe the results of the search and selection process, from the number of records identified in the search to the number of studies included in the review, ideally using a flow diagram.	Results, Section 3.1 (Lines 246–251) and Figure 1 (Line 858)
	16b	Cite studies that might appear to meet the inclusion criteria, but which were excluded, and explain why they were excluded.	Results, Section 3.1 (Lines 246–251) and Figure 1: reasons for exclusion reported (full text not found n=11; outcome indicators cannot be extracted n=23; not meeting inclusion criteria n=20; quality too low n=4; sample size too small n=7; outcome cannot be extracted or combined n=3)
Study characteristics	17	Cite each included study and present its characteristics.	Results, Section 3.1 (Lines 252–259) and Table 1 (Line 872)
Risk of bias in studies	18	Present assessments of risk of bias for each included study.	Results, Section 3.2 (Lines 260–294), Figure 2 (Line 881), and Supplementary Figure S1
Results of individual studies	19	For all outcomes, present, for each study: (a) summary statistics for each group (where appropriate) and (b) an effect estimate and its precision (e.g. confidence/credible interval), ideally using structured tables or plots.	Results, Sections 3.4–3.5 (Lines 314–411); Figure 5 forest plots (Line 895); Table 1
Results of syntheses	20a	For each synthesis, briefly summarise the characteristics and risk of bias among contributing studies.	Results, Section 3.2 (Lines 260–294); Section 3.4 (Lines 314–326) and corresponding network descriptions in

Section and Topic	Item #	Checklist item	Location where item is reported
			each secondary outcome section
	20b	Present results of all statistical syntheses conducted. If meta-analysis was done, present for each the summary estimate and its precision (e.g. confidence/credible interval) and measures of statistical heterogeneity. If comparing groups, describe the direction of the effect.	Results, Sections 3.4–3.5 (Lines 314–411); Figures 3, 4, 5, 7, 8, 9, and 10
	20c	Present results of all investigations of possible causes of heterogeneity among study results.	Results, Section 3.4 (Lines 321–326): "consistency model versus inconsistency model showed minimal difference (1.3 points)…all P-values > 0.05"; Supplementary Figure S1 (node-splitting analysis)
	20d	Present results of all sensitivity analyses conducted to assess the robustness of the synthesized results.	Results, Section 3.2 (Lines 288–294): "excluding these higher-risk studies would not be expected to materially alter the direction, magnitude, or SUCRA rankings of the main results…supported by the node-splitting analysis confirming consistency between direct and indirect evidence across all closed loops (all P>0.05)"
Reporting biases	21	Present assessments of risk of bias due to missing results (arising from reporting biases) for each synthesis assessed.	Results, Section 3.3 (Lines 300–304): "comparison-adjusted funnel plots could not be reliably constructed…potential for publication bias could not be formally assessed and was not used as a GRADE downgrading criterion, though it cannot be excluded as a source of bias"
Certainty of evidence	22	Present assessments of certainty (or confidence) in the body of evidence for each outcome assessed.	Results, Section 3.3 (Lines 295–313) and Table 2 (Line 876)
**DISCUSSION**			
Discussion	23a	Provide a general interpretation of the results in the context of other evidence.	Discussion, paragraphs 1–6 (Lines 414–499)
	23b	Discuss any limitations of the evidence included in the review.	Discussion, paragraph 7 (Lines 519–537): "sparse, star-shaped networks for pain and Apgar outcomes…extreme event sparsity for severe third/fourth-degree lacerations…substantial heterogeneity in intervention protocols…evidence base applies primarily to primiparous women…majority of included trials conducted in high-resource settings"
	23c	Discuss any limitations of the review processes used.	Discussion, paragraph 7 (Lines 519–537): "short-term follow-up precludes assessment of long-term outcomes including sexual function recovery and

PRISMA 2020 Checklist

Section and Topic	Item #	Checklist item	Location where item is reported
			development of pelvic floor disorders"
	23d	Discuss implications of the results for practice, policy, and future research.	Discussion, paragraphs 7–8 (Lines 500–548) and Conclusion section (Lines 551–569)
**OTHER INFORMATION**			
Registration and protocol	24a	Provide registration information for the review, including register name and registration number, or state that the review was not registered.	Abstract (Lines 65–66) and Methods, Section 2.1 (Lines 140–142): "prospectively registered with PROSPERO, registration number: CRD42025633266"
	24b	Indicate where the review protocol can be accessed, or state that a protocol was not prepared.	Methods, Section 2.1 (Lines 140–146): accessible via PROSPERO (https://www.crd.york.ac.uk/PROSPERO/)
	24c	Describe and explain any amendments to information provided at registration or in the protocol.	Not reported in the manuscript since no change.
Support	25	Describe sources of financial or non-financial support for the review, and the role of the funders or sponsors in the review.	Funding section (Lines 584–585): "This research received no external funding"
Competing interests	26	Declare any competing interests of review authors.	Conflict of Interest section (Lines 586–588): "The authors declare that the research was conducted in the absence of any commercial or financial relationships that could be construed as a potential conflict of interest"
Availability of data, code and other materials	27	Report which of the following are publicly available and where they can be found: template data collection forms; data extracted from included studies; data used for all analyses; analytic code; any other materials used in the review.	Data Availability Statement (Lines 570–573): "The original contributions presented in the study are included in the article/supplementary material. Further inquiries can be directed to the corresponding authors"

From: Page MJ, McKenzie JE, Bossuyt PM, Boutron I, Hoffmann TC, Mulrow CD, et al. The PRISMA 2020 statement: an updated guideline for reporting systematic reviews. BMJ 2021;372:n71. doi: 10.1136/bmj.n71
