# Supplementary material for: Comparative effectiveness of physical interventions for preventing perineal trauma during vaginal delivery: a systematic review and Bayesian network meta-analysis
Source: Front Med (Lausanne). 2026 Apr 7;13:1794056. doi: 10.3389/fmed.2026.1794056 (PMC13096049; doi:10.3389/fmed.2026.1794056)
Supplement: Supplementary file 5 [file Table_3.docx]

TableS3 Between-Study Heterogeneity Estimates (τ) for Each Outcome

| Outcomes | τMedian | τ 95% CrI | τ² |
| --- | --- | --- | --- |
| Overall Perineal Laceration | **0.413** | **0.24–0.66** | **0.170** |
| **1st-Degree Laceration** | **0.347** | **0.17–0.62** | **0.121** |
| **2nd-Degree Laceration** | **0.545** | **0.28–1.00** | **0.297** |
| **Severe Laceration (3°/4°)** | **1.145** | **0.3–2.79** | **1.311** |
| **Episiotomy** | **0.291** | **0.16–0.52** | **0.084** |
| **Intact Perineum** | **0.708** | **0.46–1.14** | **0.502** |
| Mild Perineal Pain | **1.267** | **0.36– 3.36** | **1.607** |
| **Moderate Perineal Pain** | **0.5876** | **0.24–1.10** | **0.345** |
| Severe Perineal Pain | **1.44** | **0.459–3.113** | **2.083** |
| Apgar 1-min | **0.177** | **0.011–0.399** | **0.0316** |
| Apgar 5-min | **0.153** | **0.008–0.395** | **0.023** |
